# Irreversibility in bacterial regulatory networks

**DOI:** 10.1126/sciadv.ado3232

**Published:** 2024-08-28

**Authors:** Yi Zhao, Thomas P. Wytock, Kimberly A. Reynolds, Adilson E. Motter

**Affiliations:** ^1^ Department of Physics and Astronomy, Northwestern University, Evanston, IL 60208, USA.; ^2^ Center for Network Dynamics, Northwestern University, Evanston, IL 60208, USA.; ^3^ The Green Center for Systems Biology–Lyda Hill Department of Bioinformatics, University of Texas Southwestern Medical Center, Dallas, TX 75390, USA.; ^4^ Department of Biophysics, University of Texas Southwestern Medical Center, Dallas, TX 75390, USA.; ^5^ Northwestern Institute on Complex Systems, Northwestern University, Evanston, IL 60208, USA.; ^6^ National Institute for Theory and Mathematics in Biology, Evanston, IL 60208, USA.

## Abstract

Irreversibility, in which a transient perturbation leaves a system in a new state, is an emergent property in systems of interacting entities. This property has well-established implications in statistical physics but remains underexplored in biological networks, especially for bacteria and other prokaryotes whose regulation of gene expression occurs predominantly at the transcriptional level. Focusing on the reconstructed regulatory network of *Escherichia coli*, we examine network responses to transient single-gene perturbations. We predict irreversibility in numerous cases and find that the incidence of irreversibility increases with the proximity of the perturbed gene to positive circuits in the network. Comparison with experimental data suggests a connection between the predicted irreversibility to transient perturbations and the evolutionary response to permanent perturbations.

## Teaser

Transient gene perturbations cause heritable transcriptomic changes in *E. coli*, thus one genotype can have multiple phenotypes.

## INTRODUCTION

A common goal in both statistical physics and systems biology is to connect the attributes of microscopic entities with observable macroscopic properties. Of particular interest are macroscopic properties that are emergent—including pattern formation (*1*) and synchronization (*2*)—because they arise from interactions between system entities and can therefore enable new system-level functionality. In statistical physics, an important emergent property is the irreversibility of macroscopic processes (*3*), where entropy—a state function—can increase irreversibly despite the time reversibility of the microscopic dynamics. A related property is hysteresis (*4*), where a cyclic (reversible) change of a variable leads to a persistent change in the state of the system. 

In molecular biophysics, a central dogma (*5*) posits that phenotype is determined by genotype and would thus be reversible. That is, identical DNA sequences would yield identical observable characteristics, which are assumed to arise from the proteins of the cell. The dogma allows for the possibility that multiple DNA sequences can map to the same protein amino acid sequence through synonymous codon usage, but multiple amino acids are not assigned to the same codon. Thus, rigorous adherence to this dogma cannot account for eukaryotic processes like organismal development (*6*), cell differentiation (*7*), cell reprogramming (*8*–*10*), and nongenetic aspects of aging (*11*, *12*), which may nevertheless be attributed to epigenetics (e.g., histone modifications and DNA methylation). It also does not account for environmentally induced switches in prokaryotic systems, such as stalking in *Caulobacter crescentus* (*13*), sporulation in *Bacillus subtilus* (*14*), and the *lac* and *mar* operons in *Escherichia coli* (*15*, *16*)—despite epigenetic mechanisms being largely absent in prokaryotes. The extent to which the phenotype-genotype correspondence holds for prokaryotes beyond specific cases remains an outstanding question. It is thus natural to ask how and when phenotype, which is to first approximation a state function of gene activity, can change irreversibly in prokaryotes even in response to transient microscopic (e.g., single-gene) perturbations. Here, we will consider a change to be irreversible when such a short-lived perturbation leads to a long-lasting heritable phenotypic change that persists across multiple cell divisions. In a deterministic mathematical model, these changes would be associated with transitions between stable states and hence be permanent. While dissipation is necessary for the existence of stable states, it is not sufficient for irreversible changes to occur, since the final stable state may be the same as the original one. By considering genetic rather than environmental perturbations, we can address whether transcriptional regulation itself deviates from the central dogma and does so even in the absence of environmental inducers.

**Fig. 1.**
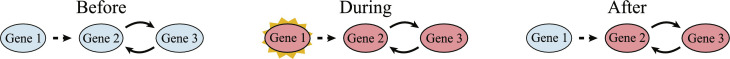
Example of the mechanism for irreversibility in a simple network of activating relationships. Before perturbation, genes are in the “OFF” state (blue color). During the perturbation, gene 1 is perturbed (yellow star background), which turns “ON” genes 2 and 3 (red). After the perturbation, gene 1 is restored to its initial OFF state, but genes 2 and 3 remain ON.

Here, we study the prevalence and network mechanisms of irreversibility in the gene regulatory network of *E. coli*, which is the model organism with the most complete reconstructed network of this kind now available. Because this reconstruction is the union of potential regulatory interactions whose presence may depend on the environmental conditions, we consider irreversibility across a range of representative sets of interactions. Using Boolean dynamics modeling, we examine the impact of transient single-gene perturbations on the activity of the other genes. We predict that transient knockouts (KOs) and transient overexpressions (OEs) of individual genes in the central part of the regulatory network commonly result in irreversible changes in the states of other genes in the network. Our results identify positive circuits (network cycles with an even number of repressive interactions) as the relevant network structures underlying the multistability necessary for irreversibility. Mechanistically, a transient perturbation may lead to permanent changes when it alters (directly or indirectly) the state of one or more multistable positive circuits (Fig. 1). This condition is more frequently satisfied the closer the perturbed gene is to a downstream positive circuit, and thus the likelihood of a gene being irreversible increases with its proximity to (or membership in) a nontrivial strongly connected component (SCC) of the network. Comparing with existing data on adaptive evolution experiments, our predictions support the hypothesis that the genes remaining in different states following adaptive evolution to a (permanent) gene KO are largely determined by those that respond irreversibly to a transient KO of the same gene. The results point to specific candidates for observing irreversibility and its presence in prokaryotes despite the tight transcription-to-translation coupling and the still largely unresolved role of epigenetic mechanisms in such organisms.

## RESULTS

### Network modeling approach

We focus our analysis on the transcriptional regulatory network from the RegulonDB database, *G*(*V*, *E*), which is reconstructed from empirical data and includes ∣*V*∣ = 1859 genes (nodes) and ∣*E*∣ = 5119 pairwise signed interactions (edges) (*17*). We retain only activating (positive sign) and repressing (negative sign) interactions, leaving out 148 edges with dual or unknown function. Recognizing that the subset of regulatory interactions present (but not their polarity) may vary depending on the cultivation conditions, we examine the average irreversibility propensity across a representative ensemble of dynamical rules formed by these interactions. To ensure that our analysis is conducted on a connected component of the network, we identified all origons. An origon is a subnetwork that starts at a root node with no incoming edges (other than autoreglatory ones) and includes all downstream nodes and edges that can be reached from the root node by following directed paths (*18*).

We analyze the largest origon, which is rooted at gene *phoB* and consists of a 1406-node subnetwork; the results would be similar for all other large origons since they are accounted for by related core networks (table S1). The *phoB* origon is also the largest subnetwork reachable from any individual node in the RegulonDB model. The origon core is the subnetwork *G*′(*V*′, *E*′) that remains after recursively removing the nodes with zero outgoing edges. According to this procedure, which generalizes the metabolic network trimming used in (*19*), the core network shares similarities with the concept of *k*-core applied to the outgoing edges. Our notion of core network is nevertheless different from other generalizations of *k*-core on directed networks (*20*) as the procedure here is tailored to capture the irreversibility of the entire origon. This is the case because nodes outside the core have no impact on the state of upstream nodes (including all nodes in the core) and have reversible impacts on the state of downstream nodes. In the case of the *phoB* origon, the core network consists of ∣*V*′∣ = 87 nodes and ∣*E*′∣ = 290 edges. Given that the number of Boolean network states scales as 2 ^∣*V*′∣^, our focus on the core also leads to a dimension reduction that helps circumvent a combinatorial explosion in simulations of the dynamics.

The network dynamics are modeled using a Boolean framework (*21*, *22*) on the core network with nodes *u* = 1, …, ∣*V*′∣ and edges *E*′ ⊆ *V*′ × *V*′. Edges are denoted by (directed) ordered pairs (*v*, *u*), indicating that the gene associated with tail node *v* regulates the gene associated with head node *u*. The polarity of (*v*, *u*), *W*(*v*, *u*), is +1 or −1 indicating activation or repression. The state of the network at time *t* is indicated by $x^{t}=\left(x_{u}^{t}\right)$, where $x_{u}^{t}\in \left\{0,1\right\}$ is the Boolean state of gene *u*. Each $x_{u}^{t}$ is assumed to evolve according to a deterministic Boolean function *B_u_* : {0,1}^*V*′^ → {0,1}

$$x_{u}^{t+1}=B_{u}\left(x^{t}\right)$$ (1)

which accounts for the $k_{u}^{+}$ edges incident on *u* in *G*′ and their polarities by obeying three network consistency constraints. The constraints are edge consistency (nodes with states on the right-hand side in Eq. 1 must connect to the node *u*), edge essentiality (all nodes on the right-hand side are necessary to determine $x_{u}^{t+1}$), and sign consistency ($x_{v}^{t}$ or ${\bar{x}}_{v}^{t}$ appears on the right-hand side if *v* activates or inhibits *u*). Here, we use that the negation of *x_v_* = 0 is ${\bar{x}}_{v}=1$, and the negation of *x_v_* = 1 is ${\bar{x}}_{v}=0$. As a consequence, *B_u_*(**x**) can be written as a sum of products of *x_v_* and/or ${\bar{x}}_{v}$ (modulus 1) for all *v* with edges incident on *u*, where *x_v_* (${\bar{x}}_{v}$) appears and does so once if the polarity *W*(*v*, *u*) is positive (negative) (*23*). For additional details and the special case of autorepression, we refer to Materials and Methods.

**Fig. 2.**
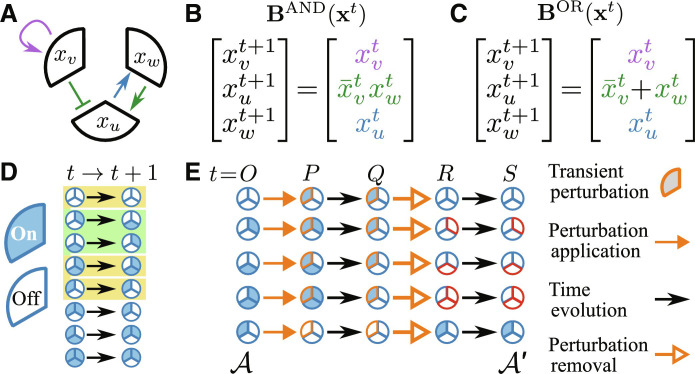
Finding irreversible perturbations in a Boolean gene regulatory network of three nodes. (**A**) Representation of a three-gene network, where the sectors correspond to genes *u*, *v*, and *w* with states *x_u_*, *x_v_*, and *x_w_*. Here, as in all subsequent network figures, pointed arrowheads indicate activating relationships, and flat arrowheads indicate repressive relationships. (**B** and **C**) Boolean functions consistent with the network edges and polarities in (A), as indicated by text colors and negations (bars), respectively. The functions **B**^AND^ and **B**^OR^—labeled according to the function assigned to update node *u*—provide rules for synchronous updates of the node states at each time *t*. (**D**) State transitions for the update rules **B**^AND^ where sector colors indicate the node state. The yellow and green backgrounds indicate fixed-point and period-2 attractors, respectively. (**E**) Application and removal of a perturbation to each attractor state. This transient perturbation can leave the network in a different attractor, with the altered node states between the initial attractor 𝒜 and final attractor 𝒜′ indicated by a red outline.

As an example of consistent update rules, consider the regulatory relationships illustrated in the three-gene network of Fig. 2A. Because node *u* has two incoming edges ($k_{u}^{+}=2$), there are two feasible functions that satisfy the network consistency constraints, $B^{\text{AND}}=\left(B_{u}^{\text{AND}}\right)$ and $B^{\text{OR}}=\left(B_{u}^{\text{OR}}\right)$ (Fig. 2, B and C, respectively). Both rules have multiple attractors 𝒜, which are fixed point or periodic orbits ${\left.\left\{x_{𝒜}^{t}\right\}\right|}_{t=1}^{T_{𝒜}}$, *T*_𝒜_ ≥ 1, to which the other states converge over time. Figure 2D shows the state transitions and attractors associated with **B**^AND^. The attractors form the starting point for identifying irreversible transient perturbations as illustrated in Fig. 2E. Starting from each attractor (*t* = *O*), each *x_u_* is perturbed independently of 1 to 0 for KOs and from 0 to 1 for OEs (*t* = *P*). The states are allowed to evolve under the perturbation until reaching a new attractor (*t* = *Q*). The perturbation is removed upon reaching the attractor (*t* = *R*), and the states evolve to the final attractor 𝒜′ (*t* = *S*). If 𝒜′ ≠ 𝒜, then we classify the transient KO or OE as an irreversible perturbation and refer to genes that differ between attractors as irreversible response genes.

**Fig. 3.**
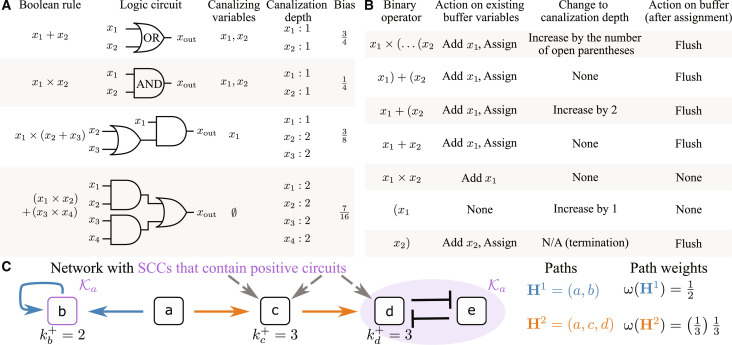
Example calculations of the key Boolean concepts underlying the network ensemble. (**A**) Examples of Boolean rules, their logic circuit representation, canalizing variables, variable canalization depth, and rule bias. (**B**) Scheme for calculating the canalization depth of the variables. Each row of the table explains how the binary operator determines the assigment of the canalization depth of the variables. N/A, not applicable. (**C**) Illustration of the path weight calculation for network paths to SCCs that contain positive circuits.

To proceed with the analysis of irreversibility in our model, we must first specify the rules of the core regulatory network. Because the number of possible rules is too large to simulate exhaustively (see Materials and Methods), we developed an algorithm to sample the ensemble of rules based on key qualities of empirical and Boolean regulatory networks. In Fig. 3A, we illustrate key properties of Boolean networks relevant to our simulations. Regulatory networks have been empirically observed to have a nested canalizing structure (*24*–*26*), which occurs when the state of one incident node determines the output regardless of the state of the remaining incident nodes. Mathematically, this condition is expressed as $x_{v}=x_{v}^{*}$ implies $B_{u}\left({\left.x\right|}_{x_{v}=x_{v}^{*}}\right)=x_{u}^{*}$ independently of the states of a set of one or more other nodes incident on *u*. As seen in the first two rows of Fig. 3A, both variables in the simple AND (×) and OR (+) are canalizing, while higher-order Boolean functions with higher levels of nesting are shown in the third and fourth rows. Here, we quantify the nestedness by calculating the expected number of variable states needed to determine the output of *B_u_*, which we refer to as the canalization depth. This is calculated by expressing the Boolean rules in simplest form using the Quine-McCluskey algorithm (*27*, *28*). Briefly, we break down the simplified rule into binary operators and read each operator from left to right while keeping track of the canalization depth and a list of variables (i.e., buffer) whose depth is to be assigned. Figure 3B provides instructions on how to update the canalization depth and list of variables when each operator is encountered. For each pair of inputs, we decide whether (i) to assign the depth to each variable in the buffer, (ii) to increase the canalization depth, and/or (iii) to empty the buffer. We also account for the rule bias, which is the probability of updating to 1, as this quantity has been shown to play a role in determining the response to perturbations in random Boolean networks (*29*, *30*).

It is therefore natural to sample the ensemble using a nestedness parameter *r* and a bias parameter *s*. Each Boolean function has a list of Boolean variables determined by the network consistency constraints leaving the $k_{u}^{+}-1$ binary operations for us to specify. We assign the nesting operator “×( ” between the variables with probability *r*. In the remaining 1 − *r* probability, we assign the + operator with probability *s* and the × operator with probability 1 − *s*. Thus, the binary operators ×, +, and ×( appear with probabilities (1 − *r*)(1 − *s*), (1 − *r*)*s*, and *r*, respectively. With these parameters defined, we can enumerate all rules of $k_{u}^{+}$ variables, assign a probability of randomly obtaining each rule, and determine the canalization depth of each variable in each rule (according to the procedure in Fig. 3B). By first averaging the canalization depth over the variables in each rule and subsequently weighting by the probability of obtaining each rule, we obtain an expression for the average canalization depth. The parameters *r* and *s* also can be used to relate our representation of the rules—which focuses on a core network of densely connected cycles and explicitly accounts for the polarity of each edge in the network—to representations that treat rule inputs as statistically independent (*31*, *32*) and/or are agnostic of edge polarities (*29*, *30*).

**Fig. 4.**
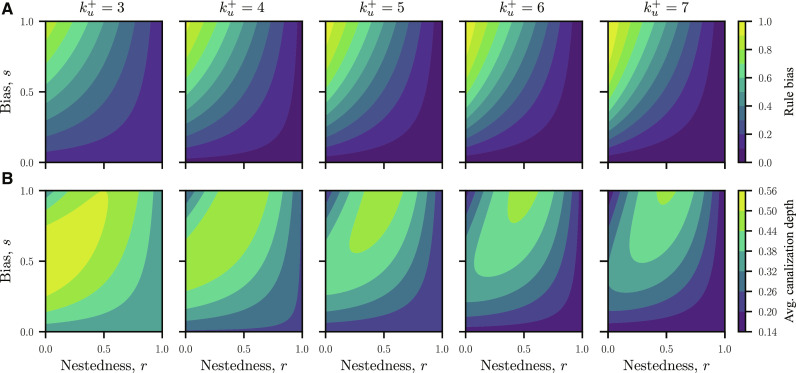
Parameter dependence of the update rules. (**A**) Rule bias and (**B**) average canalization depth for $k_{u}^{+}=3,\ldots ,7$. The rule properties are expressed as functions of the nestedness parameter *r*, which is the probability of joining two inputs with the ×( operator, and bias parameter *s*, which is the probability of joining two inputs with the + operator.

Figure 4 (A and B) shows, respectively, the rule bias and average canalization depth as functions of *r* and *s*. These quantities are determined by enumerating the $2^{k_{u}^{+}-1}$ possible input combinations and their associated probabilities. In the cases (*r* = 0, *s* = 1), (*s* = 0 ∀*r*), and (*r* = 1 ∀*s*), the rules are fully canalizing, since the first joins all pairs with a + operator and the latter two join all pairs with a × operator. The rule bias and canalizing state for each input are, respectively, $1-2^{-k_{u}^{+}}$ and 1 in the first case and $2^{-k_{u}^{+}}$ and 0 for the remaining cases. The average canalization depth reaches a maximum at the point (*r*, *s*) = (0.5, 1), where the rule bias takes an intermediate value. For fixed values of *r* and *s*, the algorithm requires the specification of an ordering of inputs. We consider two limiting cases: concentrated control, in which an incident node *v* with the largest out-degree $k_{v}^{-}$ tends to canalize the other inputs, and diffuse control, in which a node with the smallest $k_{v}^{-}$ tends to canalize the others. The former scenario is analogous to the disassortativity observed in the structure of regulatory networks, in which nodes with large *k*^−^ tend to be connected to nodes with small *k*^−^ (*33*). This situation allows for the cell’s transcriptional state to be broadly altered by changing a select few transcription factors, sometimes referred to as “general transcription factors” in bacteria (*34*) or “master regulators” in eukaryotes (*35*). Under diffuse control on the other hand, genes referred to as “specific transcription factors” in bacteria (*34*) or “secondary regulators” in eukaryotes (*35*) tend to canalize the output. This scenario distributes control of the transcriptional state across many small circuits, enabling the spatiotemporal encoding of specific responses to particular signals (*36*). Together, concentrated and diffuse control reflect competing strategies responsible for the organization of gene regulatory networks, with the latter case expected to have many more attractors than the former case. In fig. S1, we indeed observe a significantly larger number of attractors associated with networks in the latter scenario for parameters with large average canalization depth. The geometric mean over realizations ranges from the order of 10^2^ attractors for concentrated control (descending sorting) to 10^3^ attractors for diffuse control (ascending sorting).

This framework allows us to probe the irreversibility in the core network of the *phoB* origon *G*′. We generate update rules **B** of the core network for each *r* = [0, 0.2, 0.4, 0.6, 0.8, 1] with *s* = 1, and for each *s* = [0, 0.2, 0.4, 0.6, 0.8, 1] with *r* = 1 − *s* and 0. For the (*r*, *s*) pairs with nonunique rules, we sample *M* = 20 realizations in each input order (for later reference, we define *M* = 1 for unique rules). We determine the attractors for each realization of **B** using an SAT-based algorithm (*37*), finding that the number of attractors varies with the rule nestedness and input sorting. The number of attractors is largest for intermediate values of the rule nestedness—although we note that the biological relevance of a given attractor varies widely.

### Analysis of the irreversibility results

**Fig. 5.**
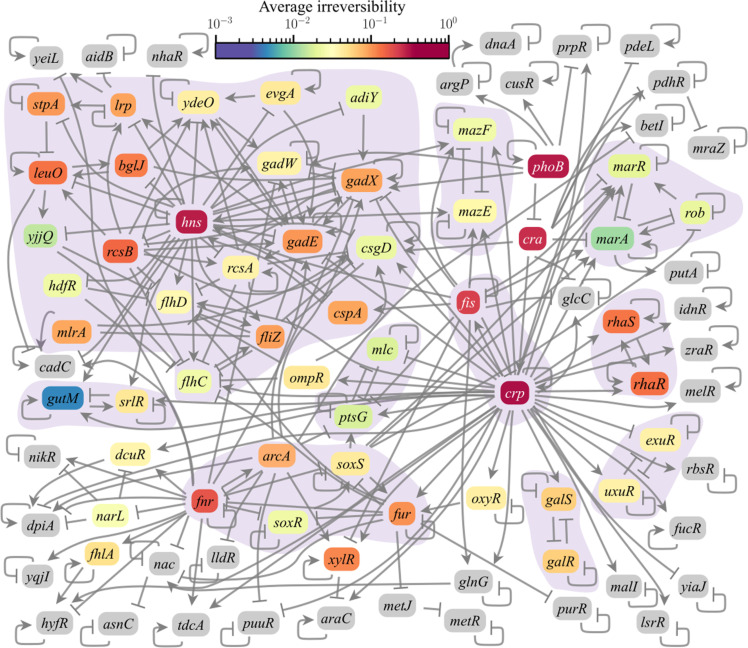
The 87-gene core regulatory network of the *phoB* origon in *E. coli*. Node colors encode the average irreversibility probability across *M* realizations of the rules, and edges denote regulatory interactions. The shaded background indicates the multinode SCCs, which all have one or more positive circuits. In total, 51 genes admit irreversible perturbations. The average irreversibility probability across realizations is within 0.1 of the true value (fig. S2).

Figure 5 summarizes the average probability that each gene in the *phoB* origon core network admits an irreversible perturbation. Because such perturbations are on nodes that cause others to change when perturbed, these nodes reside in, or upstream of, nontrivial SCCs that contain at least one circuit with positive polarity. (An SCC is by definition a subnetwork for which each node can be reached by every other node, and we define trivial SCCs as single-node SCCs with no autoregulation.) Circuits are directed loops in the network formed by a set of *m* distinct nodes and *m* distinct edges, and the circuit polarity is the product of the polarities of the edges. Positive circuits are necessary for the existence of multiple fixed-point attractors (*38*), which constitute the most common attractor class observed in our simulations. This creates the possibility of multiple stable states (*39*, *40*), which we show below is a necessary condition for irreversibility. The probability that the change is irreversible increases with proximity to downstream SCCs (shaded subnetworks in Fig. 5). Because large SCCs tend to have more positive circuits, they create more opportunities for multistability, which helps explain their observed proximity to upstream nodes admitting irreversible perturbations. At the same time, the greater complexity of large SCCs means that they likely contain both positive and negative circuits. Figure S3 shows that, in a minority of cases, this combination strengthens the irreversible response of selected genes compared to (smaller) SCCs with purely positive circuits by facilitating irreversible responses. These results are an example of the network structure playing a role in determining irreversibility.

Every node influencing a positive circuit in the network exhibits irreversibility for some **B** in our simulations. The remaining nodes cannot permanently alter the state of any positive circuit when transiently perturbed, and they are one of two types: (i) leaf nodes (i.e., nodes with no outgoing edges to different nodes), which are reversible because they cannot influence other nodes; and (ii) nodes influencing only autorepressive leaf nodes, which are reversible because the leaf node circuits are necessarily monostable. We refer to the Supplementary Materials for details and gene identities in each case. Leaf nodes can still be irreversible response genes when they are downstream of an irreversibly responding positive circuit, which illustrates that network structure also constrains the possible irreversible response genes.

To establish necessary and sufficient conditions for irreversibility, we examine the transitions induced by the application and removal of perturbations. For a perturbation node *u*, we define the state inversion operator ${\left.g_{u}\left(x\right)=x\right|}_{x_{u}={\bar{x}}_{u}}$, and we refer to the time points in Fig. 2E. The operator *g_u_* changes the state of variable *x_u_* to its inversion ${\bar{x}}_{u}$ while leaving the remaining states in **x** unchanged. To prove the necessary condition by contradiction, suppose that the perturbation of *u* is irreversible (i.e., 𝒜′ ≠ 𝒜) and that *g_u_*(**x***^Q^*) = **x***^O^*. However, by definition, *g_u_*(**x***^Q^*) = **x***^R^*, so {**x***^O^*, **x***^R^*, **x***^S^*} ⊆ 𝒜, making the perturbation reversible, a contradiction. As a consequence, there exists a nonempty set $𝒲=\left\{w|w\neq u,x_{w}^{Q}\neq x_{w}^{O}\right\}$. Irreversibility further requires the existence of some *v* ∈ *V*′ such that *B_v_*(**x***^R^*) ≠ *B_v_*[*g*_𝒲_(**x***^R^*)], where we extend the *g* operator to sets of nodes 𝒲. In the absence of such a *v*, **x***^R^* and *g*_𝒲_(**x***^R^*) belong to the same 𝒜′, and 𝒜′ = 𝒜 because *g*_𝒲_(**x***^R^*) = **x***^O^* by the definition of 𝒲. We can now state a sufficient condition for irreversibility in terms of the set 𝒲 and the basin of attraction of 𝒜, which is the set of states that reach 𝒜 for some *t* ≥ 0. The condition is that **x***^R^* cannot be in the basin of attraction of 𝒜, which implies *B_v_*(**x***^R^*) ≠ *B_v_*[*g*_𝒲_(**x***^R^*)] for some *v* ∈ *V*′. Direct inspection of our simulations confirm that these conditions are satisfied in Fig. 5.

Since the basins of attraction are relevant to the sufficiency condition for irreversibility, we recalculated a weighted average of irreversibility in which the irreversibility in each initial attractor is weighted proportional to the size of its basin (fig. S4). These weighted irreversibility results remain qualitatively similar to unweighted case, as indicated by the *R*^2^ > 0.91 for both diffuse and concentrated control input orderings. However, the rate of irreversibility is cut approximately in half, and the basin sizes of the initial attractors are on average approximately one-eighth the size of the final attractor basins. This tendency for irreversible perturbations to drive the network from attractors with smaller basins to those with larger basins can be understood as a consequence of the necessary and sufficient conditions: A set of downstream genes must change state in response to the irreversible perturbation (necessary condition), and the state reached upon reversion—provided that it is outside the original basin (sufficient condition)—is more likely to belong to a larger basin of attraction than a smaller one.

### Dynamical versus structural factors influencing irreversibility

**Fig. 6.**
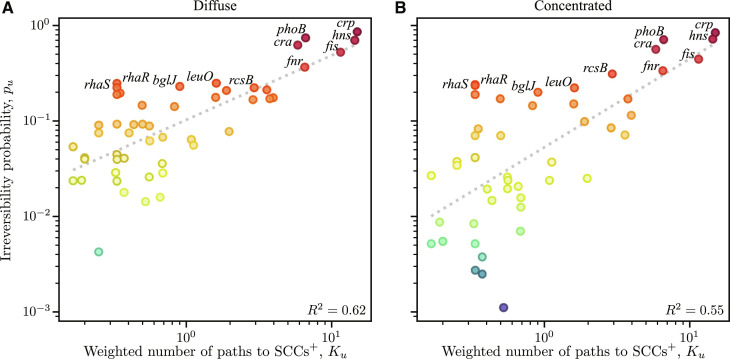
Irreversibility probability for all nodes in the *phoB* origon core network as a function of the weighted number of paths to SCCs with positive circuits (SCCs^+^). (**A** and **B**) Results for diffuse and concentrated control scenarios (i.e., ascending and descending input sorting), respectively. The best fit trend is indicated by the dotted line in each case with the indicated coefficient of determination (*R*^2^). The color code is the same as in Fig. 5.

Casting irreversibility in terms of the set 𝒲 allows us to relate *p_u_*, the average irreversibility probability across all rules of node *u* (a dynamical property), to the weighted number of paths to downstream SCCs with a positive circuit (a structural property). In Fig. 3C, we illustrate a simple network with two paths to SCCs with a positive circuit. Each path is defined as a sequence of 𝓁 ≥ 2 nodes $H^{m}=\left(H_{1}^{m},\ldots ,H_{𝓁}^{m}\right)$ (indexed by *m*), in which $\left(H_{i}^{m},H_{j}^{m}\right)\in E\prime$ and $H_{i}^{m}\neq H_{j}^{m}$ for all *i* ≠ *j*; in addition, $H_{𝓁}^{m}\in {𝒦}_{u}$, where 𝒦*_u_* is the set of nodes in all downstream SCCs of *G*′ with a positive circuit. We argue that, starting at $u=H_{1}^{m}$, each path to 𝒦*_u_* can contribute to the possibility that a perturbation gives rise to a nonempty 𝒲. We define the weight of path **H***^m^* to be $\omega \left(H^{m}\right)={\left(\prod_{i=2}^{𝓁}‍k_{H_{i}^{m}}^{+}\right)}^{-1}$. (Example calculations of the path weight are provided in Fig. 3C.) Under certain approximations, the path weight corresponds to the probability that the perturbation of *u* changes the state of nodes in 𝒦*_u_* through the path **H***^m^*. These approximations are that each input of $B_{H_{i}^{m}}$ is equally probable to change its output and that the change in state of each node in the path is independent. Now, considering all paths from a node *u* to 𝒦*_u_*, the weighted number of paths to all (other) nodes in SCCs for node *u* is *K_u_* = ∑_{**H***^m^*}_ ‍ ω(**H***^m^*). Figure 6 shows that 55 to 62% of the variance in *p_u_* in our simulations is accounted for by the relationship ${\hat{p}}_{u}=aK_{u}^{b}$, where *b* = 0.68 ± 0.09 for diffuse control and *b* = 0.93 ± 0.14 for the concentrated control. The larger exponent in the latter case reflects the reduced probability among nodes *u* with a smaller number of weighted paths to 𝒦*_u_*.

**Fig. 7.**
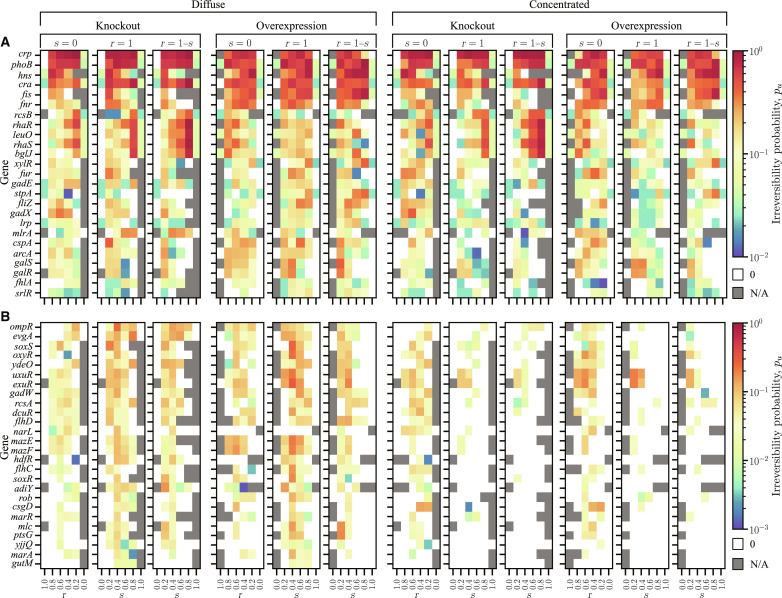
Probability of admitting irreversible perturbations averaged over realizations for the input orderings and perturbation types indicated above the panels. (**A**) Color-coded irreversibility probability as a function of *r* and *s* for the top 25 genes with the highest probability. For each input ordering and perturbation type, the three panels (from left to right) show the irreversibility probability along three different straight lines in the (*r*, *s*)-space in Fig. 4. (**B**) Same plot as (A) for the remaining 26 genes admitting irreversible perturbations. The first and last columns in each panel correspond to the cases of all inputs joined by + and all inputs joined by ×, respectively.

Figure 7 illustrates the trend in irreversibility averaged across realizations as a function of the input orderings, perturbations types, and values of *r* and *s* for the 25 most irreversible genes (Fig. 7A) and the remaining genes (Fig. 7B). Within each panel varying *s* and/or *r*, the columns are ordered such that the rule bias increases from left to right. The first and last columns are common to all panels of a given perturbation type because the rules are unique for these parameter choices. In Fig. 7A, the irreversibility of the set of genes formed by *hns*, *stpA*, *crp*, *rcsB*, *leuO*, *bglJ*, *rhaR*, and *rhaS* varies monotonically with the rule bias in all three panels for each choice of ordering and perturbation type. Specifically, from left to right, the irreversibility decreases for the KO of *hns* and the KO of *stpA*, but it increases for the KOs of the remaining genes in this set. For each of these genes, as a function of the rule bias, the irreversibility of their OEs is anticorrelated with the irreversibility of their KOs. The anticorrelation is related to the number of attractors in which a particular gene is on (or off) in a given realization of the rules; that is, a perturbed gene tends to show greater irreversibility with respect to the perturbation (KO or OE) that can be applied to the largest number of attractors. This can be intuitively understood by considering the limiting case in which only one attractor has a given gene on. In this case, any irreversible transition induced by the KO of this gene requires the perturbed gene to change state after the restoration of the KO. Such a change in state can only occur if the perturbed gene is in a circuit with other genes that change state after the initial perturbation. This is in sharp contrast with the other extreme in which the given gene is on in all attractors, and thus irreversibility is possible even if it remains unchanged after the KO is restored.

Figure 7A also shows that genes *phoB*, *cra*, and *fis* exhibit a decrease in the irreversibility probability for intermediate values of *r* and/or *s*, which may be attributed to the larger average canalization depth for these parameter values. These genes have a large number of regulatory outputs $k_{u}^{-}$ and, because of the increased canalization depth, become less likely to determine the state updates compared to genes like *fnr*, *fur*, *fliZ*, and *gadX*, which have a lower overall irreversibility probability but show an increase in this probability for intermediate values of *r* and/or *s*. Genes in the latter group tend to be located within large SCCs, while genes in the former group tend to be situated upstream of multiple SCCs. Last, Fig. 7B shows the remaining 26 genes that admit irreversible perturbations but have smaller average irreversibility probability. These genes tend to have a small out-degree ($k_{u}^{-}$), and thus they are most strongly affected by the input ordering. In the descending input ordering, the genes with large out-degree dominate those with small out-degree, resulting in the dearth of irreversibility for this ordering compared to the ascending ordering.

### Irreversible genes in adaptive responses to *crp* KO

**Fig. 8.**
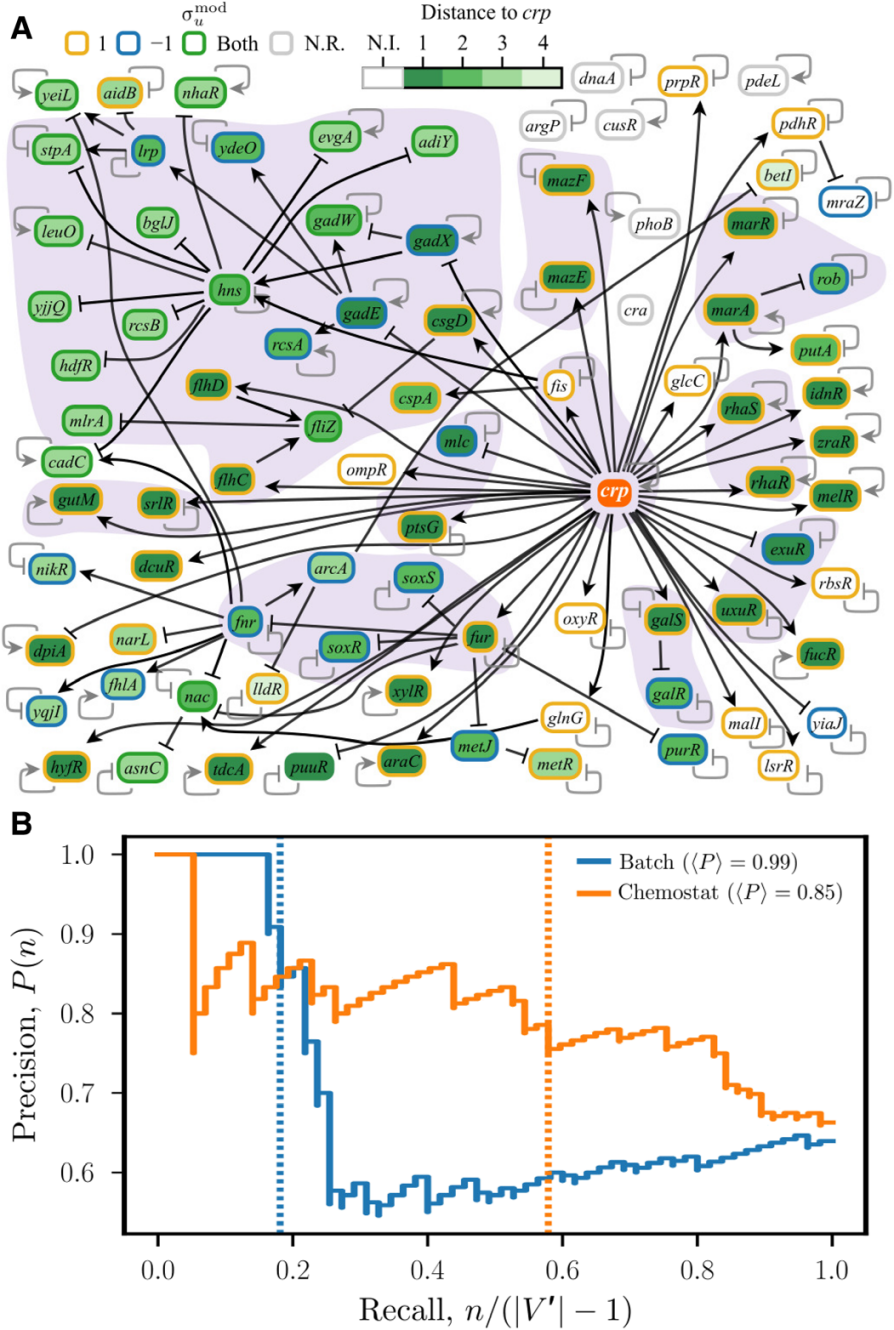
Comparison of the irreversibility results with the observed transcriptional changes in adaptive evolution. (**A**) Representation of the *phoB* origon core network showing in black the edges that appear in the shortest paths to each node from *crp*. The node outline colors indicate the sign of *crp* regulation, and the node colors indicate the distance from *crp*, where irreversible response nodes are green. The shaded backgrounds, autoregulatory edges, and network layout are the same as in Fig. 5. (**B**) Precision-recall curves evaluating the agreement of the sign of expression change of each gene predicted by the Boolean network model with that observed after adaptive evolution in batch (blue) and chemostat (orange) conditions. The genes are ranked from largest to smallest in terms of their change in expression. The dotted lines indicate the threshold of 0.5 for the log fold change used to calculate the 〈*P*〉 for each condition (marked on the legend). Genes above this threshold in batch and chemostat conditions are listed in tables S2 and S3, respectively. N.R., not regulated; N.I., not irreversible.

The irreversible responses to transient genetic perturbations predicted here have implications for adaptive evolution. Intuitively, the response of the other genes to a gene KO followed by adaptive evolution is akin to the response to a gene KO followed by its reversion—in the sense that both adaptation and the response to reversion tend to compensate for the changes induced by the initial perturbation. In Fig. 8, we examine this proposition by comparing the behavior of the genes in the core network that respond irreversibly to *crp* KO—the most irreversible perturbation in our simulations—with those that do not in terms of their transcriptional changes during adaptive evolution to this perturbation. The gene *crp* encodes the catabolite repressor protein, a global transcriptional regulator that represses genes associated with the metabolism of nonpreferred carbon sources in the presence of glucose. We make use of existing RNA sequencing (RNA-seq) data from experiments where the cells were evolved for 10 days in M9 glucose following *crp* KO, which provides the highest-quality characterization of the transcriptome under these conditions (*41*). Using these data, we compute the observed sign and magnitude of the log fold change in expression between the initial and adaptively evolved strains, which were characterized under both batch and chemostat cultivation (details in Materials and Methods). The observed sign for gene *u*, denoted $\sigma_{u}^{\mathrm{obs}}$, is compared against the sign predicted by the Boolean model $\sigma_{u}^{\text{mod}}$. The latter is the opposite of the polarity of the shortest path of *crp* to the gene (Fig. 7A). Using *u*′ to denote the genes ordered in terms of decreasing magnitude of their log fold change, we compute the precision of the top *n* genes

$$P\left(n\right)=\frac{1}{n}\sum_{u\prime =1}^{n}‍𝕀\left(\sigma_{u\prime }^{\text{obs}}=\sigma_{u\prime }^{\text{mod}}\right)$$ (2)

which is the rate at which the signs match among these genes. Here, 𝕀 is the indicator function, which takes the value 1 if its argument is true and 0 if it is false. When there are multiple shortest paths of the same length to a given gene and two of these paths have different polarities, the indicator function evaluates to 1 for nonzero values of $\sigma_{u}^{\mathrm{obs}}$. Figure 8B shows the precision *P*(*n*) for both batch and chemostat conditions plotted as a function of *n* (normalized by ∣*V*′∣ − 1, the total number of genes in the *phoB* origon core network other than *crp*). The precision decreases rapidly in both conditions when log fold change becomes less than a threshold of 0.5. Of the genes with a log fold change above this threshold, 10 of 11 in batch cultivation and 33 of 42 in chemostat cultivation change in the direction prescribed by the Boolean model. Then, the average precision, defined by

$$\langle P\rangle \left(n\right)=\frac{1}{n}\sum_{m=1}^{n}‍P\left(m\right)$$ (3)

is 0.99 and 0.85 in batch and chemostat cultivation, respectively (tables S2 and S3). Both scenarios yield a significant *P* value less than 0.01, as determined by bootstrapping (see Materials and Methods).

Having established that the correspondence between $\sigma_{u}^{\mathrm{obs}}$ and $\sigma_{u}^{\text{mod}}$ is statistically significant, we examine the extent to which the genes with $\sigma_{u}^{\text{obs}}=\sigma_{u}^{\mathrm{mod}}$ and a log fold change >0.5 also corresponded to the 68 irreversible response genes associated with *crp* KO in the Boolean model. We find that 8 of 9 irreversible response genes match the predicted response compared to 2 of 2 reversible genes in batch culture, and 28 of 34 irreversible response genes match response compared to 5 of 8 reversible genes in chemostat culture, yielding a *P* value of 0.03 when considering both conditions together (see Materials and Methods). It is notable that a statistically significant relationship between the predicted irreversibility and adaptive evolution experiments is detected despite the limited information on the actual regulatory rules in the Boolean model and the nonregulatory factors known to influence adaptive evolution.

### Making specific predictions

Motivated by the concordance between the gene expression changes during adaptive evolution, we examine the irreversible perturbation of *crp* KO in the context of existing transcriptional data and more detailed models of gene regulation (fig. S5). First, we calculate whether each gene responds irreversibly to *crp* KO across all attractors for all realizations of the rules. We assess the biological relevance of the attractors by weighting the irreversibility results based on the similarity of each attractor to the observed transcriptional states when calculating the average irreversibility (fig. S6). This analysis leads us to conclude that self-activating genes that are positively regulated by *crp* are the most likely to be irreversible.

While the level of detail in the Boolean model allows us to determine the type of perturbation (KO) and the initial states of the genes (both ON), it does not provide us with information regarding the continuous dynamics of the gene expression. We obtain a continuous version of the Boolean dynamics that preserves the stable states by using the HillCube algorithm (*42*) to represent the Boolean AND regulation as a differential equation. In fig. S7A, we use this representation to calculate the conditions for multistability in terms of phenomenological parameters like the transcriptional activation strength and Hill coefficient (a measure of how step-like the activation rule is). The irreversibility predictions from this analysis are verified by simulating the equations (fig. S7B). From these simulations, we infer qualitative aspects that enhance irreversibility in this motif: Irreversible response genes will tend to have stronger self-activation and exhibit a more switch-like response (i.e., have a larger Hill coefficient).

Overall, this analysis suggests candidate irreversible response genes such as *zraR*, *melR*, and *rhaRS* in response to *crp* KO. These genes are convenient because they one can ensure that they are initially on by adjusting the cultivation conditions (e.g., by using glyercol which is known to activate *crp* and by supplementing the growth media metabolites such as zinc, melibiose, or rhamnose in the cases of *zraR*, *melR*, and *rhaRS*, respectively). Meanwhile, one candidate method for implementing the transient KO is inducible CRISPR interference (*43*). Last, expression of the response genes could be monitored via sequencing to ascertain whether irreversibility occurs. Comparing with experiments exploring bistability in inducible sugar utilization (*44*), we posit that there will be a range of modest concentrations of supplemental metabolites the irreversible response gene will turn off, corresponding to the bistable region in fig. S7A.

## DISCUSSION

The irreversibility of transient gene regulatory perturbations predicted here reveals a mechanism for prokaryotic cells to exhibit distinct phenotypes even when they are genetically identical. Our analysis, which excludes extracellular factors and chromatin modifications from the model, emphasizes the ability of purely regulatory mechanisms to precipitate heritable nongenetic changes that can endure for multiple generations. This should be compared with the phenomenon of cell fate commitment in eukaryotes, which is typically attributed to an environmental factor or signaling molecule triggering the expression of a master regulator that orchestrates the activation and repression of downstream genes to achieve a change in phenotype (*45*, *46*). In eukaryotes, epigenetic modifications such as histone modification and DNA methylation play a role in locking cells into their fates (*47*, *48*), but the former process is absent and the latter functions differently in prokaryotes.

Within the scope of our *E. coli* model, we establish that genes admitting irreversible perturbations rely on positive circuits to generate multistability and stabilize their irreversible responses. This finding reveals greatly enhanced complexity in the repertoire of possible cell states, well beyond those previously observed for bistable chemosensory motifs (*15*, *16*, *44*). Together, the results lead to the interesting possibility of nongenetically programming the state of bacterial cells—a phenomenon ultimately related to the control aim of steering between attractors in the regulatory network (*49*–*51*). Broadening our model to account for stochastic fluctuations, cell cycle, and other nonstationary factors can convert the permanently irreversible responses seen in our model into temporarily irreversible but long-lived changes that persist over multiple generations. The extent to which the predicted irreversibility will persist and be inheritable is thus an important question for future experimental studies, which can be interpreted using stochastic many-body physics approaches tailored to describe the processes of transcription, translation, and degradation (*52*–*57*).

Finally, our analysis suggests that genes responding irreversibly are significantly associated with those that undergo regulatory changes in adaptive evolution experiments across conditions, even in the absence of full knowledge of the regulatory rules. This result is consistent with the observation that incomplete models of gene regulatory networks can still yield reliable predictions (*32*). Thus, notwithstanding the simplifications of the model, the analysis of irreversible responses to transient perturbations also contributes to the interpretation of adaptive evolution responses to permanent perturbations.

## MATERIALS AND METHODS

### Construction of the *phoB* origon core network

We constructed the activating and repressing interactions of the gene regulatory network based on the RegulonDB data using the file “generegulation_tmp.txt” (*17*), where pairwise regulatory relationships between genes are recorded. The regulatory network dynamics were simulated using R (version 4.2.3) and R package BoolNet (version 2.1.8) (*58*). For the dynamics to be well defined, each node must have at least one input, where we recognize the rule $x_{u}^{t+1}=x_{u}^{t}$ as positive autoregulation. Therefore, in analyzing the *phoB* origon, we added a self-activating loop (and no additional inputs) to *phoB* as this is the only gene in the core network with no regulatory inputs. This added edge fixes the initial state of the node and does not affect our observation of irreversibility.

### Generation of biologically realistic update rules

Because the available RegulonDB data are insufficient to specify all the Boolean update rules, we examine the ensemble of consistent rules. Rules are said to be consistent with RegulonDB if they satisfy the three criteria laid out in the main text:

1) Edge consistency. The state variables *x_v_* (or their negation ${\bar{x}}_{v}$) appearing on the right-hand side of Eq. 1 are those associated with nodes *v* that have edges incident on *u* in *G*′.

2) Edge essentiality. Whenever *v* is a node incident on *u* in *G*′, there is at least one state **x** for which changing the variable *x_v_* changes *B_u_*(**x**).

3) Sign consistency. We require that *B_u_*(**x**_*x_v_*=0_) ≤ *B_u_*(**x**|_*x_v_*=1_) if *v* activates *u* and *B_u_*(**x**_*x_v_*=0_) ≥ *B_u_*(**x**|_*x_v_*=1_) if *v* inhibits *u*.

To exclude artifactual oscillations, we further assume that autorepressive regulation is silenced when *x_u_* = 0 in the sign consistency condition, which implies an exception to the edge essentiality condition. Specifically for every rule chosen, one or more edges into a autorepressive node will not influence the state of this node. If the autorepressive node is in its own monomial, the self-edge is nonessential. If the autorepressive node is joined with others in a monomial, then the other input edges to this node in the monomial will be nonessential.

### Estimating the number of possible regulatory rules

Edge and sign consistency together guarantee that one of $x_{v}^{t}$ or ${\bar{x}}_{v}^{t}$ appears in the rule *B_u_*, and edge essentiality guarantees that all *v* must appear once. Thus, the number of variables in the rule *B_u_* is $k_{u}^{+}$. Since all possible Boolean rules can be written as a sum of products (*23*), the number of feasible rules is at least as large as $\sum_{n=0}^{k_{u}^{+}-1}‍\left(\begin{array}{c} k_{u}^{+}-1\\ n \end{array}\right)=2^{k_{u}^{+}-1}$. Because the *B_u_* are set independently for each *u*, the number of feasible **B** is $\prod_{u=1}^{|V\prime |}‍2^{k_{u}^{+}-1}$, which of the order of 10^61^ for the *phoB* origon core network.

### Algorithm to sample realistic update rules

In the general case, given a network, there is an ensemble of possible **B** that are consistent with the network structure and polarity of the interactions. To facilitate the sampling of this ensemble, we introduce the vector of inputs $y=\left[y_{v_{1}},\ldots ,y_{v_{k_{u}^{+}}}|\left(v_{i},u\right)\in E\prime \right]$ for fixed *u*, where *y_v_i__* = *x_v_i__* if *W*(*v_i_*, *u*) > 0 and $y_{v_{i}}={\bar{x}}_{v_{i}}$ if *W*(*v_i_*, *u*) < 0. The inputs are indexed using $i\in \left\{1,\ldots ,k_{u}^{+}\right\}$ and ordered according to $k_{v_{i}}^{-}$, the number of outgoing edges of the associated node *v_i_*, for both ascending ($k_{v_{i}}^{-}\leq k_{v_{i+1}}^{-}$) and descending ($k_{v_{i}}^{-}\geq k_{v_{i+1}}^{-}$) orders. The sampling of the ensemble of rules is parameterized by *r* and *s*, which control the selection among three binary operators between inputs *y_v_i__* and *y*_*v*_*i*+1__. Specifically, we join the inputs as *y_v_i__* × (*y*_*v*_*i*+1__ with probability *r*, as *y_v_i__* + *y*_*v*_*i*+1__ with probability (1 − *r*)*s*, and as *y_v_i__* × *y*_*v*_*i*+1__ with probability (1 − *r*)(1 − *s*). Larger values of the nestedness parameter increase the number of parentheses appearing in the rules, and larger values of the bias parameter increase the number of possible input vectors that update to 1. We specify each *B_u_* by starting at the first pair of inputs, choosing their binary operator according to the probabilities above, and proceeding iteratively until all inputs are included. This strategy is implemented in the following iterative algorithm:


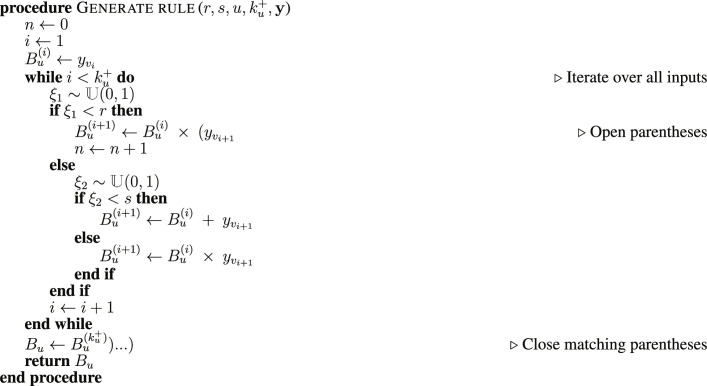


In the algorithm, we use ∼𝕌(0,1) to denote a random number drawn from the uniform distribution on the unit interval, and we use )…) to denote the *n* closed parentheses in the rule.

### Convergence of irreversibility estimates

For each pair (*r*, *s*) and input sorting that does not have unique rules [*r* ≠ 1,  *s* ≠ 0, and (*r*, *s*) ≠ (0,1)], we generate *M* = 20 realizations of the rules indexed by *i* ∈ {1, …, *M*}, identify the attractors (Fig. 2D), and apply the irreversibility detection algorithm (Fig. 2E). Since the attractors change between realizations of the rules, the irreversibility of a perturbation (KO or OE) may also change. To account for this source of variability, we average over the transient perturbations as follows. Let *q*_*u*,*i*_ be the fraction of attractors for which *x_u_* = 1, and let $p_{u,i}^{\text{KO}}$ and $p_{u,i}^{\text{OE}}$ be the probabilities that the transient KO and OE of gene *u* lead to irreversibility, respectively. Then

$${\hat{p}}_{u}^{𝒮}=\frac{1}{|𝒮|}\sum_{i\in 𝒮}‍p_{u,i}^{\text{KO}}q_{u,i}+p_{u,i}^{\text{OE}}\left(1-q_{u,i}\right)$$ (4)

is the weighted average of the probability that gene *u* admits an irreversible perturbation across a set of realizations 𝒮.

We test for the convergence of the average irreversibility as a function of the ensemble size by fixing the number of realizations to be *M*′ = {1, …, 10}. The number of possible ensembles of size *M*′ taken out of *M* realizations is $\left(\begin{array}{c} M\\ M\prime \end{array}\right)$, and the number of pairs of ensembles is $Z\left(M,M\prime \right)=\left(\begin{array}{c} M\\ M\prime \end{array}\right)\left(\begin{array}{c} M-M\prime \\ M\prime \end{array}\right)$. If *Z*(*M*, *M*′) > 1000, then we randomly sample 1000 pairs of ensembles. Otherwise, we use all *Z*(*M*, *M*′) pairs. Denoting each ensemble pair as (𝒰, 𝒱), we apply Eq. 4 to each ensemble to obtain the root mean square difference

$$\text{RMSD}=\sqrt{\frac{1}{|V\prime |}\sum_{u=1}^{|V\prime |}‍{\left({\hat{p}}_{u}^{𝒰}-{\hat{p}}_{u}^{𝒱}\right)}^{2}}$$ (5)

where we recall that ∣*V*′∣ = 87 is the number of genes in the core network.

### Processing of the RNA-seq data

The transcriptional data for *E. coli* adaptively evolved after *crp* KO are obtained and analyzed as follows. Raw counts of RNA were obtained from the Gene Expression Omnibus (GEO) database (*59*) maintained by the National Center for Biotechnology Information (NCBI), accession number GSE152214. Experimental details of the RNA collection have been described elsewhere (*41*). Raw counts in GEO were converted into transcripts per million using $z_{i}=10^{6}\frac{c_{i}}{L_{i}}{\left(\sum_{j=1}^{N_{g}}‍\frac{c_{j}}{L_{j}}\right)}^{-1}$, where *N_g_* is the number of genes in the dataset, *c_i_* is the raw count of transcripts for gene *i*, and *L_i_* is the length of the gene in kilobases. The transcript counts of genes that are in the core regulatory network *G*′ in our model were examined for changes before *crp* KO, after *crp* KO, and after adaptive evolution of the *crp* KO strain. The data include strains cultivated under both batch and chemostat conditions.

### Calculating the observed sign of the transcriptional changes

For each environmental condition, $\rho_{u}=\mu_{u}^{\text{evo}}/\mu_{u}^{\text{wt}}$ is the fold change of the average expression for each gene between the adaptively evolved strain and the initial wild-type strain. To account for the overall shift in transcription (e.g., due to changes in the lab conditions or variability in media preparation), we calculate the average shift in expression $\langle \rho \rangle ={\left|V\prime \right|}^{-1}\sum_{u=1}^{|V\prime |}‍\mu_{u}^{\text{evo}}/\mu_{u}^{\text{wt}}$. Then, the observed sign of regulatory changes is

$$\sigma_{u}^{\text{obs}}=\text{sgn}\left(\text{ln}\frac{\rho_{u}}{\langle \rho \rangle }\right)$$ (6)

where sgn(ϵ) is the sign function that takes the value 1 if ϵ > 0, −1 if ϵ < 0, and 0 if ϵ = 0. In addition, the magnitude of the log fold change in expression is ∣ln(ρ*_u_*/〈ρ〉)∣.

### Calculating the predicted sign of the transcriptional changes

The predicted sign of the transcriptional change in the Boolean model is assigned according to the polarity of the shortest paths in *G*′ from *crp* to each gene *u*. Recall that the a shortest path between $H_{1}^{u}=\mathrm{crp}$ and $H_{𝓁}^{u}=u$ is denoted by $H^{u}=\left(H_{1}^{u},\ldots ,H_{𝓁}^{u}\right)$ and that the polarity of the edges is given by the function *W*. According to the Boolean model, the sign of the expected change is

$$\sigma_{u}^{\text{mod}}=-\prod_{i=1}^{𝓁-1}‍W\left(H_{i}^{u},H_{i+1}^{u}\right)$$ (7)

where the negative sign appears because the perturbation of *crp* is a KO. Equations 6 and 7 provide the quantities used in calculating the precision in Eq. 2.

### Statistical analysis of 〈*P*〉 and irreversibility

We assess statistical significance using a bootstrapping approach (*60*). In this approach, we compute *N*_exc_, the number of times that the randomized list returns a larger value of the statistic than the observed lists out of *N*_samp_ = 25,000 shufflings, and the *P* value is given by 1 − *N*_exc_/*N*_samp_. Specifically, we compute 〈*P*〉 in each condition to evaluate whether the concordance between $\sigma_{u}^{\text{obs}}$ and $\sigma_{u}^{\text{mod}}$ is statistically significant. Using *u*′ to denote the reordered list of genes, 〈*P*〉 is repeatedly computed after shuffling $\sigma_{u\prime }^{\text{mod}}$ while keeping $\sigma_{u}^{\text{obs}}$ and ∣ln(ρ*_u_*/〈ρ〉)∣ fixed.

Similarly, we compute the number of genes that satisfy $\sigma_{u}^{\text{obs}}=\sigma_{u}^{\text{mod}}$ and ∣ln(ρ*_u_*/〈ρ〉)∣ > 0.5 for genes responding irreversibly (γ_irr_) and reversibly (γ_rev_) to *crp* KO in each cultivation condition. Using the difference γ_irr_ − γ_rev_ in each condition, we assess whether genes that show large changes during adaptive evolution are significantly more likely to be irreversible in our simulations. In this case, we repeatedly compute γ_irr_ − γ_rev_ after shuffling $\sigma_{u\prime }^{\text{mod}}$ while keeping $\sigma_{u}^{\text{obs}}$ and ∣ln(ρ*_u_*/〈ρ〉)∣ fixed in each condition separately. We count cases toward *N*_exc_ only when γ_irr_ − γ_rev_ exceeds the observed value in both environmental conditions.

## Supplementary Materials

### This PDF file includes:

Supplementary Text Figs. S1 to S7 Tables S1 to S3 References
