# Supplementary material for: Irreversibility in bacterial regulatory networks
Source: Sci Adv. 2024 Aug 28;10(35):eado3232. doi: 10.1126/sciadv.ado3232 (PMC11352831; doi:10.1126/sciadv.ado3232)
Supplement: Supplementary file 1 — Supplementary Text Figs. S1 to S7 Tables S1 to S3 References [file sciadv.ado3232_sm.pdf]

Science Advances
AAAS

Supplementary Materials for

Irreversibility in bacterial regulatory networks

Yi Zhao *et al.*

Corresponding author: Adilson E. Motter, motter@northwestern.edu

Sci. Adv. **10**, eado3232 (2024)
DOI: 10.1126/sciadv.ado3232

This PDF file includes:

Supplementary Text
Figs. S1 to S7
Tables S1 to S3
References

Summary

This Supplementary Material file contains details regarding (i) the consistency of the irreversibility estimates across realizations, (ii) a characterization of the nodes not exhibiting irreversibility, (iii) a discussion of the necessity of positive circuits for irreversibility, (iv) conditions necessary for the preservation of synchronous partial fixed points under asynchronous updates, (v) an examination of the network motifs responsible for certain periodic attractors, (vi) a comparison with alternative formulations of the ensemble of rules, (vii) a comparison with previous studies investigating bacterial heterogeneity, (viii) a comparison of adaptive evolution responses to *crp* KO with irreversible response genes in the Boolean model, and (ix) a detailed description of how to use our results to obtain specific experimental predictions.

Irreversibility across realizations of the update rules

We demonstrate that the averages for the irreversibility estimated from different samplings of the rules converge to a common value. Figure S2 presents the distributions of the RMSD for all considered parameter pairs with nonunique rules for ascending and descending input sortings (i.e., diffuse and concentrated control) in panels A and B, respectively. The plots in each row are arranged in order of decreasing rule bias [cf., Fig. 4A]. Importantly, neither the rule bias nor the average canalization depth appears to alter the convergence. We observe that the RMSD converges to approximately 0.1 in probability for two independent ensembles of $M' = 10$ realizations, with slightly higher values observed in ascending order compared to descending order. These results establish that $M = 20$ realizations are sufficient to obtain reliable estimates of the average irreversibility. In the main text, the ensemble averages over realizations are compared with irreversibility for unique rules, which do not require averaging.

In addition to considering the impact of update rules, we also examined the impact of changing the point of the attractor in which the perturbation was reverted in the case of periodic attractors. Throughout the paper, we assess irreversibility by applying the perturbation to the first point of each attractor recorded by the SAT algorithm (*37*). Similarly, we revert the perturbation in the first point of the perturbed trajectory that is on the new attractor. We argue that the impact of this choice is small, which we confirm by comparing with the irreversibility observed when reverting the perturbation of the closest point of the new attractor to the initial state in terms of Hamming distance. We find that reverting from this state causes 2.1% of irreversible transitions to become reversible.

Nodes not exhibiting irreversibility

The algorithm that generates the core network G' recursively trims nodes that are trivial SCCs, so that the core network contains all irreversible perturbations. This is the case because if all inputs of an SCC consisting of a single node v with no autoregulation remain unchanged after a transient perturbation, then x_v must remain unchanged as well. Thus, it can be concluded that the trimming will not alter the number of fixed points in the network, which is consistent with known necessary conditions for multi-stationarity (*38–40, 61*). Now focusing on the core, Fig. 5 shows that 36 nodes within G' do not admit irreversible perturbations across all realizations of the rules in our simulations. We examine these nodes on a case-by-case basis and show that they cannot be irreversible by directly demonstrating that the final state must belong to the same attractor as the initial state. There are two cases:

1. *Reversibility of leaf nodes.* Consider a leaf node u, which by definition has no edges to other nodes. Such a node is necessarily reversible because it does not influence other nodes and is restored to its original state upon removal of the transient perturbation. A total of 32 nodes are in this class—*aidB*, *araC*, *asnC*, *betI*, *cadC*, *cusR*, *dnaA*, *dpiA*, *fucR*, *glcC*, *glnG*, *hyfR*, *idnR*, *lldR*, *lsrR*, *malI*, *melR*, *metR*, *mraZ*, *nhaR*, *nikR*, *pdeL*, *prpR*, *purR*, *putA*, *puuR*, *rbsR*, *tdcA*, *yeiL*, *yiaJ*, *yqjI*, and *zraR*.

2. *Reversibility of nodes exclusively regulating autorepressive leaf nodes.* The genes *metJ*, *pdhR*, *argP*, and *nac* have a single regulatory output to an autorepressive leaf node. Either the regulatory node canalizes the output of the leaf node or the leaf node's autoregulatory input (which is by definition 0) overrides the other input. In the former case, any change to the state of the leaf node caused by the perturbation of the regulatory node is restored by its reversion, whereas in the latter case the state of the leaf node never changes.

Necessity of positive circuits for irreversibility

In the main text, we focus on positive circuits and claim that these circuits underlie the multistability necessary for irreversibility. Here we justify this claim. *We first observe that the edge consistency condition implies the existence of at least one circuit in the network.* The edge consistency condition requires that each node in the network has an incident edge (this assumes the existence of autoregulatory edges in nodes without input edges from other nodes). It follows that in a connected component of $|V|$ nodes, starting at an arbitrary node and

following incident edges backwards $|V|$ times, we necessarily visit a node more than once. This implies the existence of a circuit.

We next observe that circuits are necessary for multistability and, by implication, for irreversibility. This is because nodes not belonging to circuits are trivial SCCs and thus are not irreversible response nodes according to the argument in the previous section. The *phoB* origon core network has numerous positive circuits, guaranteeing multiple stable attractors.

Irreversibility occurs between two attractors. To orient the discussion, we note that in our simulations, 42.2% of the attractors are period-1 attractors, 24.4% are period-2 attractors, 1.1% are period-3 attractors, 27.9% are period-4 attractors, and 4.4% are higher-period attractors. For brevity, we use the language *periodic attractors* to refer to period-n attractors with $n > 1$, while we continue to use *fixed points* to refer to period-1 attractors. Irreversibility may involve a transition between two fixed-point attractors, two periodic attractors, or a fixed-point attractor and a periodic attractor.

We first consider the case of irreversibility arising from a transition between two fixed-point attractors, noting that 49.0% of transitions in our simulations are of this type. *We observe that fixed-point attractors cannot arise from negative circuits.* In a network consisting of a negative circuit, the fixed-point condition $\mathbf{x} = \mathbf{B}(\mathbf{x})$ implies that the state x_v would be negated an odd number of times in the process of applying the update rules (*38*), which would require that $x_v = \bar{x}_v$ (we recall that the overbar indicates negation). Since this is impossible, no fixed-point attractors can exist for a network consisting of a negative circuit. In the absence of positive circuits, application of the fixed-point condition to a network including any number of negative circuits will yield one or more contradictions of the form $x_v = \bar{x}_v$, which again exclude the possibility of fixed-point attractors. In the absence of positive circuits, the exceptions are: (i) negative loops consisting of a single node with an autorepressive edge, which is fixed but monostable, and (ii) negative circuits that remain fixed due to inputs from upstream nodes, which precludes the possibility of multistability. Thus, irreversibility between fixed points requires positive circuits.

We note that periodic attractors in which *all* nodes are time-dependent are absent in our simulations. Thus, transitions in which one or more attractors are periodic involve *partial* fixed points, which we define as time-dependent attractors in which the state of one or more nodes are time-independent. A total of 31.7% of irreversibility transitions in our simulations occur between partial fixed-point attractors with all differences taking place among the time-independent nodes. An additional 2.2% involve a transition between a fixed-point

and a partial fixed-point attractor. The remaining transitions (17.1%) occur between partial fixed-point attractors with different numbers of fixed nodes, meaning that a subset of the time-independent nodes transition to being time-dependent and/or vice versa. In all cases, the differences between attractors involve a node that is time-independent in at least one of the attractors. We observe that the arguments of the previous paragraph apply in the sense that positive circuits determine the time-independent nodes in partial fixed-point attractors. Thus in all transitions, the attractors are determined (at least in part) by positive circuits.

We expect the time-independent portions of the initial and final attractors observed in our simulations to be preserved under asynchronous updates in the large majority of cases. This is the case because fixed-point attractors are preserved under asynchronous updates (*62*), implying that the initial and final states in the 49.0% of transitions between fixed-point attractors will exist in asynchronous updates as well. In addition, we expect the time-independent nodes of partial fixed points to be preserved in a further 16.2% of transitions, because a synchronous partial fixed point has a corresponding asynchronous partial fixed point under the conditions discussed below. Finally, the initial and final attractors in transitions involving one fixed point and one partial fixed point are preserved, and they are guaranteed to remain distinct when the partial fixed point has time-independent nodes whose state differs from those of the fixed point. The latter accounts for 2.6% of all transitions. Together, 67.9% of transitions for synchronous updates have the initial and final attractors preserved under asynchronous updates.

The time-dependent portions of partial fixed-point attractors associated with negative circuits show acyclic behavior under asynchronous updates. This is because the periodicity of an attractor generally requires the concurrent update of multiple nodes at each time step, while only one (randomly chosen) node is updated at a time under asynchronous updates. However, we did not observe transitions between attractors in which the only difference occurs between time-dependent nodes (i.e., attractors that share the same set of time-independent nodes and that have identical states on these nodes) in our simulations.

Preservation of synchronous partial fixed points

Asynchronous partial fixed points imply the existence of synchronous partial fixed points (*63*) but not vice versa. Concerning the forward implication, the states of time-independent nodes of asynchronous partial fixed points are, by definition, guaranteed to remain fixed for all possible states of the time-dependent nodes, and thus under synchronous updates. The reverse implication does not hold because the asynchronous updates

cause the time-dependent nodes to reach states outside of their synchronous periodic orbit, making it possible for the downstream time-independent nodes to change.

Notwithstanding, we identify a set of sufficient conditions under which synchronous partial fixed points have corresponding asynchronous partial fixed points. For the state of a time-independent node in a synchronous partial fixed point to change under asynchronous updates, the node must have more than one upstream input that is time-dependent. Otherwise, the time evolution on the attractor is sufficient to conclude that the time-independent state will be stable for all possible values of the time-dependent nodes. The requirement for multiple time-dependent inputs is established by as follows. We refer to the set of time-dependent nodes in the attractor as $\mathcal{B}^0$ and the set of nodes incident on u in G' as $\mathcal{I}_u$. Then, the set of nodes with at least 2 incident nodes in $\mathcal{B}^0$ is $d\mathcal{B}^0 = \{u \,|\, 1 < |\mathcal{I}_u \cap \mathcal{B}^0|\}$. Starting at $i = 0$, we set $\mathcal{B}^{i+1}$ to $\mathcal{B}^i \cup d\mathcal{B}^i$, increment i by 1, and iterate the calculation until $d\mathcal{B}^i$ is empty. Thus, the time-independent nodes not in $\mathcal{B}^i$ are preserved under asynchronous updates. As a corollary, this implies that $|d\mathcal{B}^0| = 0$ is a sufficient condition to conclude that synchronous partial fixed points imply the existence of corresponding synchronous partial fixed points. This condition is met in 82.5% of the period-2 attractors, 88.9% of the period-3 attractors, 14.4% of the period-4 attractors, and 37.6% of the higher-period attractors of our simulations. Together, the time-independent nodes of partial fixed-point attractors are guaranteed to be preserved under asynchronous updates in at least 46.3% of cases. (This is calculated by multiplying the frequency of each periodic attractor times its rate of preservation and normalizing by the total number of periodic attractors). If the initial and final attractors in irreversible transitions were uncorrelated, then 21.4% of transitions between partial fixed-point attractors would be preserved under asynchronous updates. We find that 51.2% of transitions between partial fixed points have initial and final attractors that preserved in our simulations. This percentage applies to the 31.7% of transitions between partial fixed points for which both the initial and final states have the same set of time-dependent nodes, yielding a total of 16.2% of all transitions as referenced above.

Motifs generating periodic attractors

The analysis of partial fixed points motivates us to investigate the network structures behind the period-2 and period-4 attractors, which together account for over half of the attractors in our simulations. We attribute the prevalence of period-2 and period-4 attractors specific motifs in the network. Period-2 attractors occur mainly among gene pairs that exhibit mutual negative feedback such as (*galR*, *galS*), (*mlc*, *ptsG*), (*exuR*, *uxuR*), and

(*mazE*, *mazF*). Period-4 attractors occur among gene pairs in which the first represses the second and the second activates the first, and one of the genes has no autoregulation, as in the cases of (*arcA*, *fnr*) and (*hns*, *cspA*). These pairs can have a period-4 orbit $(1,0) \to (0,0) \to (0,1) \to (1,1) \to (1,0)$ that cascades to downstream nodes.

Comparison with alternative formulations

Here, we offer evidence that the conclusions of the paper apply to the transcriptional regulatory network of *E. coli* in general, even though we focus specifically on the *phoB* origon (the largest origon) and canalizing rules. Specifically, we examine how changing the origon, the rules, and/or the mathematical formulation of the dynamics would affect the results.

Different origons. In Table S1, we report the statistics of all origons with core size ≥ 30 in our RegulonDB model (the remaining origons have core sizes ≤ 5). For our purposes, the most relevant measures are the overlaps of the core *phoB* origon with those of the alternative origons, as well as the overlaps of nodes in the nontrivial SCCs of the *phoB* origon with those of the alternative origons. As demonstrated in the main text, the ability of a node to admit an irreversible perturbation is related to its proximity to SCCs with positive circuits (in the general case, nontrivial SCCs can be made up of positive and/or negative circuits). Our model shows that most such SCCs of the largest origons are contained within the *phoB* origon core network (see overlap column in Table S1). Accordingly, the identity and irreversibility probability of nodes that admit irreversible perturbations in our analysis of the *phoB* origon core network are inclusive of almost all cases that would be found when considering all largest origons.

Threshold-based rules. One alternative to canalizing dynamics is threshold-based dynamics (*31*), in which the update rules for each node are

$$x_u^{t+1} = \begin{cases} 1 \text{ if } \sum_{i=1}^{k_u^+} y_{v_i}^t / k_u^+ > \chi_u, \\ 0 \text{ otherwise,} \end{cases} \tag{8}$$

where $\chi_u \in [0,1]$ are activation thresholds. Specializing to the case of a uniform threshold $\chi_u = \chi$, the limit $\chi \to 0$ corresponds to the case $(r,s) = (0,1)$ (i.e., all inputs joined by OR operators) whereas the limit $a \to 1$ corresponds to $(r,s) = (0,0)$ (i.e., all inputs joined by AND operators). The sorting of inputs y_{v_i} by $k_{v_i}^-$ when determining canalization also has a relationship to threshold dynamics with heterogeneous a_u. In particular,

if $\chi_{v_i} \propto k^-_{v_i}$, then nodes with small $k^-_{v_i}$ will be easier to activate. The ease of activation of these nodes makes this case analogous to the ascending input sorting in the limit that $s \to 1$ when $r = 0$. Other heterogeneous schemes with χ_u drawn uniformly from $[0, 1]$ would correspond most closely to the cases of $(r, s) = (0.4, 1)$ and $(r, s) = (0.6, 1)$ in our simulations.

Bias-based rules. Bias-based rules are a formulation of random Boolean networks in which the update rules are generated by randomly assigning 1 (with probability ν) or 0 to each possible input vector. The parameter ν is equivalent to the rule bias used in our analysis. This formulation is mathematically convenient for examining the effect of topology on the stability of Boolean networks (*29, 30*). Bias-based rules apply in the case of tree-like graphs in which the activation probabilities of nodes may be regarded as independent, and they do not rely on information about the polarity of interactions. In this work, we operate in the opposite limit: we focus specifically on the core network, where the coregulation of nodes produces correlated activations, and our empirical network allows us to explicitly incorporate edge polarity into the model.

Alternative dynamical formulations. We argue that the predictions of the model apply beyond the case of Boolean rules that are synchronously updated. Related conclusions are expected for asynchronous updates since fixed points are known to be preserved (*62*) and, as shown above, most partial fixed-points are also preserved under asynchronous update rules. Similar conclusions apply to continuous representations of the dynamics because the rules can be transformed from a discrete to a continuous representation of the state space and time that preserves the stable steady states (*42*). We examine the outcomes of such a transformation for a simple system below in an effort to develop experimental predictions that are as specific as possible. Given the correspondence between our results and continuous systems, it is natural to conjecture that transient perturbations may lead to irreversibility in stochastic models of gene regulation that account for the transcription, translation, and degradation (*52–57*). This conjecture may be investigated by mapping the irreversible perturbations in our simulations into corresponding parameter changes in the stochastic models.

Comparing with bacterial differentiation and environmental perturbation

It is instructive to compare the irreversibility from *transient genetic perturbations* with bacterial differentiation processes, such as sporulation in *B. subtilus* (*14*), stalking in *C. crescentus* (*13*), and life-cycle stages in pathogenic *E. coli* (*64*). The results can also be compared with observations of bacterial heterogeneity such as

persistence (*65*) across multiple bacterial species, the mucoid phenotype of *P. aeruginosa* (*65*), competence in *B. subtilus* (*14*), and metabolic shifts in *E. coli* in the case of the *lac* operon (*15*) and other inducible sugars (*44*). In these examples, genetically identical strains can exhibit diverse phenotypes due to *environmental cues*, and these phenotypes can persist hysteretically after the environmental cues are removed. While these examples involve environmental changes, our analysis shows that regulatory switching alone is sufficient to alter the state of positive feedback loops, resulting in irreversibility in the transcriptional state of the cell. Conversely, by relating our analysis with these examples, it follows that the transcriptional irreversibility we characterize can give rise to large phenotypic changes, including morphological and behavioral ones. This transcriptional irreversibility may be leveraged to design synthetic bacterial circuits that generate irreversible changes, emulating recent developments in mammals (*10*).

Comparison with adaptive evolution responses

In the main text, we explore the question of how similar the set of irreversible response genes to the transient perturbation of *crp* KO is to the set of genes exhibiting altered expression in the adaptive evolution response to the same KO. In Tables S2 and S3, we list the identity of the genes with the largest shifts in expression ($\ln \rho_u / \langle \rho \rangle$) when the adapted strain is cultivated in batch and chemostat conditions, respectively. To facilitate a comparison between our model and the data, we include the sign of regulation of *crp* (σ_u^{mod}), which is supposed to have the *opposite* sign as the shift in expression. As noted in the main text, 10 of 11 genes in batch cultivation and 33 of 42 genes in chemostat cultivation have changes in expression consistent with the regulatory model. The majority of these genes are found to respond irreversibly in our model. Of the genes that do not, most are directly regulated by *crp* and negatively autoregulated (*malI*, *lsrR*, *glcC*, *prpR*, *rbsR*, and *glnG*), two are negatively autoregulated but indirectly regulated by *crp* (*metR* and *purR*), and two are not regulated by *crp* (*pdeL* and *cra*). Changes in the first two cases may be due to constitutive activity of the gene promoters, which is not accounted for by our model. The final case may reflect changes to the intracellular environment not captured by the model. The sign of the gene expression responses to adaptive evolution are significantly concordant with those predicted by the model (see main text). In addition, the set of genes is enriched for irreversible response genes to *crp*.

Strategy to identify specific experimental conditions

Our Boolean framework, presented in the main text, fits into a broader strategy to identify specific conditions under which irreversibility occurs. An experiment to observe irreversibility requires the specification of (i) a candidate irreversible gene perturbation, (ii) an associated irreversible response gene, and (iii) specific cultivation conditions. Figure S5A summarizes how the scope of potential experiments narrows. Then, the flow chart in Fig. S5B explains how the various levels of modeling (which themselves are based on different kinds of input data) narrow the scope. With (i)–(iii) specified, the designed experiment would employ CRISPR-interference to carry out the gene perturbation and quantitative polymerase chain reaction (qPCR) or RNA-seq to measure the expression irreversible response gene at different time points.

Overview

One major outcome of the Boolean modeling is the prominence of *crp* KO as a perturbation that leads to irreversibility. (The fact that targeted KOs have fewer externalities on the use of cellular resources than OEs encouraged us to focus specifically on transient *crp* KO.) This motivated us to examine the Sequencing Read Archive (SRA) for *E. coli* experiments in which *crp* is perturbed. We found 16 instances of experiments characterizing *crp* downregulation either as a result of genetic perturbations or environmental shifts. Given this information, we examined the attractors that admit irreversibility of *crp* KO to determine whether they were similar to experimentally observed states, as described in the subsection on transcription-weighted attractor analysis. Briefly, predictions of irreversible response genes associated with attractors that were more similar to experimentally observed states were given higher weights than those that were more divergent. The outcome of the this analysis was a set of autoexcitatory genes that are themselves positively regulated by *crp*. This is similar to the three-gene schematic in Fig. 1, except that the two-gene positive feedback loop is reduced to a single autoexcitatory gene. Note that from the Boolean modeling, we already know that an AND operator is required for irreversibility (i.e., expression of both genes is required for activation). This requirement specifies the form of the differential equation model needed to model the candidate irreversible genes, as discussed in the subsection on transcriptional differential equation modeling. From these differential equations, it is possible to identify relative values of the parameters necessary for irreversibility to occur and thereby specify the cultivation conditions needed to observed irreversibility.

Transcription-weighted attractor analysis

Methods: We searched the GEO database (*59*) for all "Expression profiling by high-throughput sequencing" assays conducted in "*Escherichia coli*" on November 15, 2022, which yielded 495 datasets. Of these, we retained datasets that (i) had at least 10 RNA-seq samples and (ii) were conducted on non-enterohemorrhagic *E. coli*, yielding 155 datasets with 5,147 gene expression profiles. We retrieved the raw RNA-seq data from SRA, aligned it to the MG1655 genome (NCBI Reference: NC_000913.3) using Rockhopper (*66*), and calculated the TPM. The TPM calculations excluded alignments to ribosomal RNA genes.

We next extracted the TPM counts for the 87 genes in the core network and converted them to log-scale using the formula $\zeta_i = \log_{10}(z_i + 10^{-10}) + 10$, where i is an index over genes. We subsequently identified 16 instances in which *crp* downregulation was measured within a dataset. For each case, we calculated the average expression of each gene before ($t = O$) and after ($t = Q$) *crp* downregulation. The average expression was binarized to 1 if it was larger than the gene's median in the entire dataset and 0 otherwise. Next, we compared the observed expression states before and after *crp* downregulation with the attractors generated in our Boolean model before and after *crp* KO. As in the Boolean modeling, we use $\mathbf{x}$ to represent the binarized expression obtained from data. Furthermore, we use x_i^{obs} and x_i^{att} to denote the attractors obtained from experimental observations and our Boolean modeling, respectively, and we define ϕ_i to be the fraction of cases for which $x_i = 1$ in the expression data. Then, the similarity between observed and modeled states is quantified using the Hamming similarity

$$D(\mathbf{x}^{\text{obs}}, \mathbf{x}^{\text{att}}) = \exp\left(-\sum_{i=1}^{|V'|} |x_i^{\text{obs}} - x_i^{\text{att}}|\right) \tag{9}$$

and likelihood of x_i^{obs} matching x_i^{att} if each x_i is an independently assigned Bernoulli random variable with parameter ϕ_i:

$$E(\mathbf{x}^{\text{obs}}, \mathbf{x}^{\text{att}}, \boldsymbol{\phi}) = \prod_{i=1}^{|V'|} \theta_i(x_i^{\text{obs}}, \phi_i)^{\kappa_i(x_i^{\text{obs}}, x_i^{\text{att}})}, \qquad \text{where} \tag{10}$$

$$\theta_i(x_i^{\text{obs}}, \phi_i) = \begin{cases} \phi_i & \text{if } x_i^{\text{obs}} = 1, \text{ and} \\ 1 - \phi_i & \text{if } x_i^{\text{obs}} = 0, \end{cases} \qquad \text{with}$$

$$\kappa_i(x_i^{\text{obs}}, x_i^{\text{att}}) = \begin{cases} 1 & \text{if } x_i^{\text{obs}} = x_i^{\text{att}}, \text{ and} \\ -1 & \text{if } x_i^{\text{obs}} \neq x_i^{\text{att}}. \end{cases}$$

The irreversible responses are averaged using Eqs. (9) and (10) according to the following procedure:

(i) Identify the set of all attractors across all rule realizations in which $x_{crp} = 1$, denoted $\mathcal{C}^{\mathrm{on}}$, and indexed by $j \in \{1, ..., |\mathcal{C}^{\mathrm{on}}|\}$.

(ii) Characterize the genes' responses to *crp* KO in binary matrices $\Delta \in \{0,1\}^{|\mathcal{C}^{\mathrm{on}}|\times|V'|}$ and $\Psi \in \{0,1\}^{|\mathcal{C}^{\mathrm{on}}|\times|V'|}$ where $\Delta_{j\ell} = 1$ if the ℓth gene responds irreversibly in attractor j and $\Psi_{j\ell} = 1$ if the ℓth gene responds reversibly in attractor j.

(iii) For each instance of *crp* downregulation, indexed by $k \in \{1, ..., 16\}$, fix $\mathbf{x}^{(k,O)}$ and $\mathbf{x}^{(k,Q)}$ and calculate the attractor weights $A_{jk} = \prod_{t\in\{O,Q\}} D(\mathbf{x}^{(k,t)}, \mathbf{x}^{(j,t)})$ or $A_{jk} = \prod_{t\in\{O,Q\}} E(\mathbf{x}^{(k,t)}, \mathbf{x}^{(j,t)}, \boldsymbol{\phi})$.

(iv) With k still fixed, normalize the A_{jk} to $\tilde{A}_{jk} = A_{jk}/\sum_{j=1}^{|\mathcal{C}^{\mathrm{on}}|} A_{jk}$ and compute the average probability of irreversible and reversible response weighted by the kth conditions, which are given by $I_{k\ell} = \sum_{j=1}^{|\mathcal{C}^{\mathrm{on}}|} \tilde{A}_{jk}\Delta_{j\ell}$ and $R_{k\ell} = \sum_{j=1}^{|\mathcal{C}^{\mathrm{on}}|} \tilde{A}_{jk}\Psi_{j\ell}$, respectively.

(v) Compute $\langle I_\ell \rangle = \sum_{k=1}^{16} I_{k\ell}$ and $\langle R_\ell \rangle = \sum_{k=1}^{16} R_{k\ell}$, the average of $I_{k\ell}$ and $R_{k\ell}$, respectively, across conditions.

Using these steps, we calculated the weighted probability of responding reversibly and irreversibly for each gene.

Results: Figure S6 summarizes the results of the probability for each gene to respond irreversibly when weighting the attractors by their similarity to observed transcriptional states. The horizontal axis indicates the fraction of *crp* KO for which each gene changed state between the initial attractor and the attractor reached after *crp* KO. Given that the gene changed, the vertical axis reports the fraction of instances that it remained altered in the final attractor. Comparing Fig. S6A to Fig. S6B reveals that the patterns of irreversibility as they relate to network structure are mostly preserved across the two weighting strategies. In particular, genes likely to respond irreversibly lie in the top right of each plot and are highlighted by a pink box. All such genes exhibit the key motif in which *crp* activates the target gene (*X*), which also activates itself. This result motivates an exploration of more granular differential equation models covered in the next section.

In the remaining sectors of Fig. S6, we see the irreversibility as it relates to other regulatory architectures. In the lower right, we see a group of autorepressive genes activated by *crp* that turn off upon *crp* KO, but are restored with *crp* expression. Such genes would be candidates for negative controls in a high throughput

experiment, since they are likely to respond, but not likely to be irreversible. Moreover, as the distance of the candidate irreversible response genes to *crp* increases, the probability of changing upon *crp* KO decreases. This occurs because the response to the KO depends on a larger number of regulatory rules. At the same time, once a change occurs, the probability that it is irreversible becomes less concentrated around 0 and 1. This may be attributed to the larger number of ways to fit the candidate gene inside or downstream of a positive circuit for a given distance to *crp*, regulatory sign, and self-regulation.

Transcription differential equation modeling

Analysis: The key motif identified in Fig. S6 and the Boolean modeling together imply that irreversibility for the *crp* KO requires that both *crp* and *X* be present for *X* to be activated (i.e., an AND operator). To transform this Boolean logic into a set of continuous differential equations, we use the multivariate polynomial interpolation method (*42*) and make the following approximations: (i) transcription is fast relative to translation, (ii) dilution dominates protein degradation as a mechanism for protein loss, and (iii) the basal transcription rate of gene X is negligible. This yields the following system of equations for the expression of *crp* (C) and the downstream gene (X):

$$\frac{dC}{dt} = \alpha + \beta_C \frac{C^{\eta_C}}{K_C^{\eta_C} + C^{\eta_C}} - \delta C \tag{11}$$

$$\frac{dX}{dt} = \beta_X \left(\frac{C^{\eta_C}}{K_C^{\eta_C} + C^{\eta_C}} \right) \left(\frac{X^{\eta_X}}{K_X^{\eta_X} + X^{\eta_X}} \right) - \delta X, \tag{12}$$

where

1. α is the basal transcription rate of *crp*,
2. β_C and β_X are transcriptional activation parameters (resulting from transcription factor binding),
3. δ is the dilution rate,
4. η_C and η_X are Hill coefficients (which designate more switch-like behavior as they become larger), and
5. K_C and K_X are the concentrations for half-maximal activation.

These phenomenological parameters are measurable by targeted experiments, and as such, they may guide the choice of gene X. Here, we assume for simplicity that the impact of the basal transcription of X is negligible compared to the regulatory activation. This assumption does not impact the following analysis.

For the purposes of analyzing the parameters that lead to irreversibility, it is convenient to rescale the variables and parameters using

$$\bar{\alpha} = \frac{\alpha}{K_C} \quad \bar{\beta}_C = \frac{\beta_C}{K_C} \quad \bar{\beta}_X = (1 + \bar{C}^{-\eta_C})\frac{\beta_X}{K_X} \quad \bar{C} = \frac{C}{K_C} \quad \bar{X} = \frac{X}{K_X}. \tag{13}$$

Substitution of Eq. (13) into Eqs. (11) and (12) yields the following rescaled equations

$$\frac{d\bar{C}}{dt} = f_C(\bar{C}) = \bar{\alpha}_C + \bar{\beta}_C \left(1 + \bar{C}^{-\eta_C}\right)^{-1} - \delta\bar{C} \tag{14}$$

$$\frac{d\bar{X}}{dt} = f_X(\bar{X}, \bar{C}) = \bar{\alpha}_X + \bar{\beta}_X(1 + \bar{X}^{-\eta_X})^{-1} - \delta\bar{X}, \tag{15}$$

where we have suppressed the dependence of $\bar{\beta}_X$ on $\bar{C}$ in the second equation. We note that these equations yield biologically meaningful solutions when $\bar{C} > 0$ and $\bar{X} > 0$, since these variables represent concentrations of proteins in the cell. In addition, the rate parameters $\bar{\alpha}$, $\bar{\beta}_C$, $\bar{\beta}_X$, and δ as well as the concentrations K_C and K_X are all larger than zero.

We proceed to analyze the fixed points of this system of equations and their stability. Because gene X does not regulate gene C, the existence and stability of fixed points for C will not depend on X. Furthermore, the results for Eq. (14) will be immediately applicable to Eq. (15), since $\bar{C}$ will be time-independent at a fixed point. For these reasons, we suppress the subscript C in the following analysis to simplify the notation, and we emphasize that $\bar{C}$ is interchangeable with $\bar{X}$.

We proceed by setting $f(\bar{C}) = 0$ to obtain a polynomial equation for the fixed points of $\bar{C}$:

$$\left(\bar{\alpha} + \bar{\beta} - \delta\bar{C}\right)\bar{C}^{\eta} = \delta\bar{C} - \bar{\alpha}. \tag{16}$$

This equation has one or three solutions for biologically meaningful values of the parameters. By setting $df/d\bar{C} = 0$, we obtain the condition

$$\left(\bar{\alpha} + \bar{\beta} - \delta\bar{C}\right)\eta\bar{C}^{\eta-1} - \delta\bar{C}^{\eta} = \delta. \tag{17}$$

When this equation is satisfied simultaneously with Eq. (16) a pitchfork bifurcation occurs at a critical value $\bar{C}^*$.

This critical value may be obtained from the following steps:

(i) adding $\delta\bar{C}^{\eta_C}$ to each side of Eq. (17),

(ii) dividing the result by Eq. (16) to obtain a self-consistent equation for $1 + \bar{C}^{\eta_C}$ in terms of $\bar{C}$, and

(iii) using the self-consistency result to substitute out terms with $\bar{C}^{\eta_C}$ in favor of $\bar{C}$ to obtain a quadratic equation in $\bar{C}$.

The resulting equation is

$$\frac{1}{2}(\bar{C}^{*})^{2} - \frac{2\bar{\alpha}\eta_C + \bar{\beta}(\eta_C - 1)}{2\delta\eta_C}\bar{C}^{*} + \frac{\bar{\alpha}(\bar{\alpha} + \bar{\beta})}{2\delta^{2}} = 0, \tag{18}$$

which has roots at

$$\bar{C}^{*} = \frac{2\bar{\alpha}\eta + \bar{\beta}(\eta - 1)}{2\delta\eta}\left(1 \pm \sqrt{1 - \frac{\bar{\alpha}(\bar{\alpha} + \bar{\beta})}{\left(2\bar{\alpha}\eta + \bar{\beta}(\eta - 1)\right)^{2}}}\right). \tag{19}$$

For a given value of $\eta > 1$, there is a critical value of $\bar{\alpha}$ and $\bar{\beta}$ where multistability arises. This occurs when f, $df/d\bar{C}$, and $d^2 f/d\bar{C}^2$ all equal zero for the same value of $\bar{C}$. Setting $d^2 f/d\bar{C}^2 = 0$, we obtain

$$\frac{d^2 f}{d\bar{C}^2} = 0 = \frac{\bar{\beta}\bar{C}^{\eta-2}\eta\left(-\bar{C}^{\eta}\eta - \bar{C}^{\eta} + \eta - 1\right)}{\left(1 + \bar{C}^{\eta}\right)^{3}}, \quad \text{which implies}$$

$$\bar{C}_{\text{crit}} = \left(\frac{\eta - 1}{\eta + 1}\right)^{\frac{1}{\eta}}. \tag{20}$$

Notably, $\bar{C}_{\text{crit}}$ is independent of $\bar{\beta}$. Substituting this value of $\bar{C}$ into $df/d\bar{C} = 0$ yields

$$\frac{df}{d\bar{C}} = 0 = -\frac{\bar{\beta}\bar{C}^{2\eta}\eta}{\bar{C}\left(\bar{C}^{\eta} + 1\right)^{2}} + \frac{\bar{\beta}\bar{C}^{\eta}\eta}{\bar{C}\left(\bar{C}^{\eta} + 1\right)} - \delta\bar{\beta}, \quad \text{which reduces to}$$

$$\bar{\beta}_{\text{crit}} = \frac{4\eta\delta}{(\eta - 1)^{\frac{\eta-1}{\eta}}(\eta + 1)^{\frac{\eta+1}{\eta}}}, \tag{21}$$

after substituting in Eq. (20) for $\bar{C}$. Substituting Eqs. (20) and (21) into Eq. (16) results in

$$\bar{\alpha}_{\text{crit}} = \frac{(\eta - 1)^{2}}{4\eta}\bar{\beta}. \tag{22}$$

With these critical values determined, we can determine the region of ($\bar{\alpha}$,$\bar{\beta}$,η)-space that allows for multiple stable fixed points.

Figure S7A illustrates the regions of parameter space for which the system is multistable. These regions are determined by fixing η, calculating the critical point, then progressively increasing $\bar{\beta}$. For each value of $\bar{\beta}$, we adjust $\bar{\alpha}$ from $\bar{\alpha}_{\text{crit}}$ to $\bar{\alpha}_{\text{crit}} - (\bar{\beta} - \bar{\beta}_{\text{crit}})\left(1 + \bar{C}_{\text{crit}}^{-\eta}\right)^{-1}$ in $f(\bar{C})$. Next, we solve for the concentrations $\bar{C}^{+}$ and $\bar{C}^{-}$ that maximize and minimize $f(\bar{C})$ by finding the roots of $f'(\bar{C})$. Then, we compute $f(\bar{C}^{+})$ and

$f(\bar{C}^-)$ with the adjusted value of α. This procedure yields the largest and smallest values of $\bar{\alpha}$ for this value of $\bar{\beta}$, which are

$$\bar{\alpha}_{\text{max}}(\bar{\beta}) = \bar{\alpha}_{\text{crit}} - (\bar{\beta} - \bar{\beta}_{\text{crit}}) \left(1 + \bar{C}_{\text{crit}}^{-\eta}\right)^{-1} - f(\bar{C}^-), \text{ and} \quad (23)$$

$$\bar{\alpha}_{\text{min}}(\bar{\beta}) = \bar{\alpha}_{\text{crit}} - (\bar{\beta} - \bar{\beta}_{\text{crit}}) \left(1 + \bar{C}_{\text{crit}}^{-\eta}\right)^{-1} - f(\bar{C}^+). \quad (24)$$

Since only $\bar{\alpha} \geq 0$ are physically meaningful, we constrain $\bar{\alpha}_{\text{min}} \geq 0$. We also plot the critical points (determined from output Eq. (22) and Eq. (21)) parametrically as a function of $\eta \in [1, 10]$. From this analysis, it is possible to identify parameters suitable for realizing irreversibility as envisioned by the Boolean model, as shown next.

Simulations: Figure S7B shows an example of a transient knockout of *crp* causing the irreversible response of gene $\bar{X}$ from simulation of Eqs. (14) and (15). The initial parameters for *crp* and the response gene are indicated in Fig. S7A by the bold green and purple circles, respectively (the colors correspond to the value of η in the legend). By Eq. (13), the regulatory activation for $\bar{X}$ already includes the impact of *crp* regulation. This choice of parameters has a single fixed point for $\bar{C}$, which is highly expressed. The high expression of $\bar{C}$ coupled with the high initial expression of $\bar{X}$ cause the system to evolve toward a state with high concentrations of both genes. When *crp* is subsequently knocked out (implemented by decreasing $\bar{\beta}$ to 0.1 as indicated by the faded green circle in Fig. S7A), the gene concentrations evolve toward a fixed point with both concentrations near their (low) $\bar{\alpha}$. Upon restoration of the regulatory activation of *crp*, its concentration increases. The concentration $\bar{X}$, however, remains low because there is a fixed point for Eq. (15) with a low $\bar{X}$. Although this is only one choice of parameters, it is clear that there many possible choices that yield concentration trajectories similar to those observed in Fig. S7B.

Examples of potential experiments

The results of the differential equation modeling help refine the selection of candidate irreversible perturbations and irreversible response genes by allowing us to evaluate their probability of exhibiting irreversibility using qualitative knowledge about the activation strength. From the transcriptional data we collected, it appears that *crp* has a fairly large constitutive activation since it remains expressed at detectable levels even when *E. coli* is cultivated with glucose as the primary carbon source (which is known to inhibit *crp*). A larger constitutive activation restricts the values of regulatory activation (and Hill coefficient) that allow *crp* to exhibit

multistability (Fig. S7A). Since we want *crp* expression to be restored after the KO is removed, *monostability* of Eq. (14) is desirable so that $\bar{C}$ will spontaneously increase once $\bar{\beta}$ is increased. Concerning the response gene X, irreversibility requires it to have parameters that admit multistability when *crp* is active. This is facilitated by smaller constitutive activations, larger regulatory activations, and larger Hill coefficients. In most cases, larger values of the regulatory activation and Hill coefficient for *crp* also facilitate multistability. Under these conditions, the drop in expression in *crp* due to the KO causes a drop in expression of the response gene and restoration of *crp* expression fails to restore expression of the response gene as explained in Fig. S7B.

From these considerations, one can imagine an experiment in which initially high *crp* expression is achieved by cultivating *E. coli* in glycerol. The regulated gene may also be manipulated to be highly expressed, for example by including zinc in the cultivation conditions to ensure that expression of *zraR* is initially large as well. Then, the reduction in regulatory activation of *crp* can be achieved by inducing CRISPR-interference. Subsequently un-inducing CRISPR should allow the *crp* expression to recover. However, if the concentration of zinc is low enough, the regulatory activation of *zraR* can be tuned to be in the multistable region. Because *zraR* expression should be low during the induction of CRISPR, it should not be able to recover even though *crp* expression does.

One key assumption in Eqs. (11) and (12) is that the regulatory logic is qualitatively similar to a Boolean AND operator. Although this information is difficult to ascertain from the literature, it may be possible to infer cases in which the regulation is more likely to be AND based on the location of the transcription factor binding sites to DNA. If these sites are overlapping, then it is less likely that the regulatory logic is AND, whereas if *CRP* and the regulated protein form a dimer, the logic is more likely to be AND. If the regulatory rule is qualitatively an OR function, then Eq. (12) must be changed accordingly. Irreversibility may still be possible, but this would require employing an OE perturbation.

As shown in Fig. S7A, genes with stronger self-activation and larger Hill coefficients (i.e., more switch-like behavior) tend to have wider regions of multistability. Thus, the literature can be consulted to identify irreversible response gene candidates that possess these attributes. We also note that several genes may have activation parameters that can be altered via the inclusion of specific metabolites (e.g., zinc for the case of *zraR* above). The inclusion of these chemicals may be tuned to obtain a regulatory activation strength that admits multistability and to ensure that the response gene is initially ON. An analogous strategy has been previously employed to show multistability in inducible sugar utilization (*44*).

Finally, the response may be heterogeneous among cells. Such heterogeneity could occur, for example, as a result of the stochastic allocation of cellular components during cytokinesis. Since single-cell sequencing techniques in bacteria are still in their infancy, other approaches, like super-resolution fluorescent microscopy techniques, would be required to observe a heterogeneous response. The considerations in this section successfully narrow the scope of candidate irreversible perturbations and their associated response genes, but still leave substantial work to develop the required molecular biology tools implement the transient perturbation, ascertain the nature of the regulation, and detect the expression in time.

Supplementary Figures

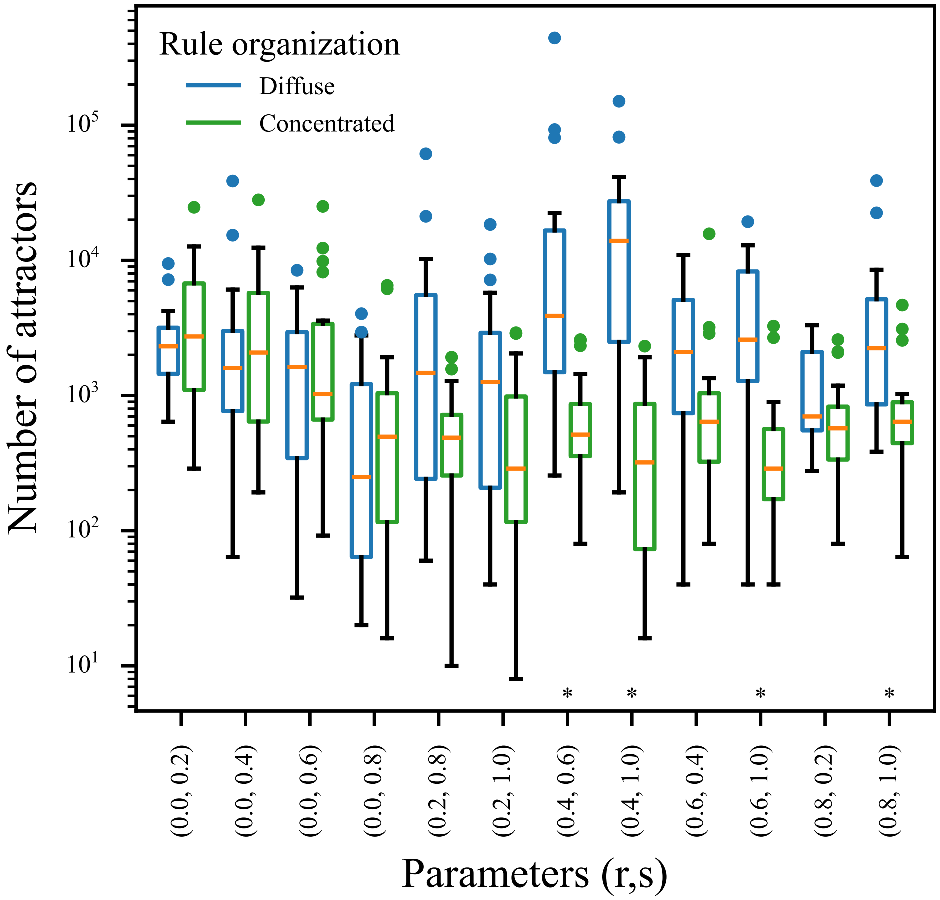

Fig. S1 Number of attractors across realizations. Boxplots of the number of attractors across the $M = 20$ realizations compare the number under the diffuse (blue) and concentrated (green) rule organization. Asterisks indicate parameters (r, s) for which the diffuse organization has a signficantly larger number of attractors as quantified by the Kruskal-Wallis test with a Bonferroni-corrected p-value < 0.004. The boxes, orange lines, upper whiskers, and lower whiskers denote the interquartile range, median, maximum (excluding fliers), and minimum, respectively. Fliers, which are data points that are more than 1.5 times the interquartile range above the median, are plotted separately.

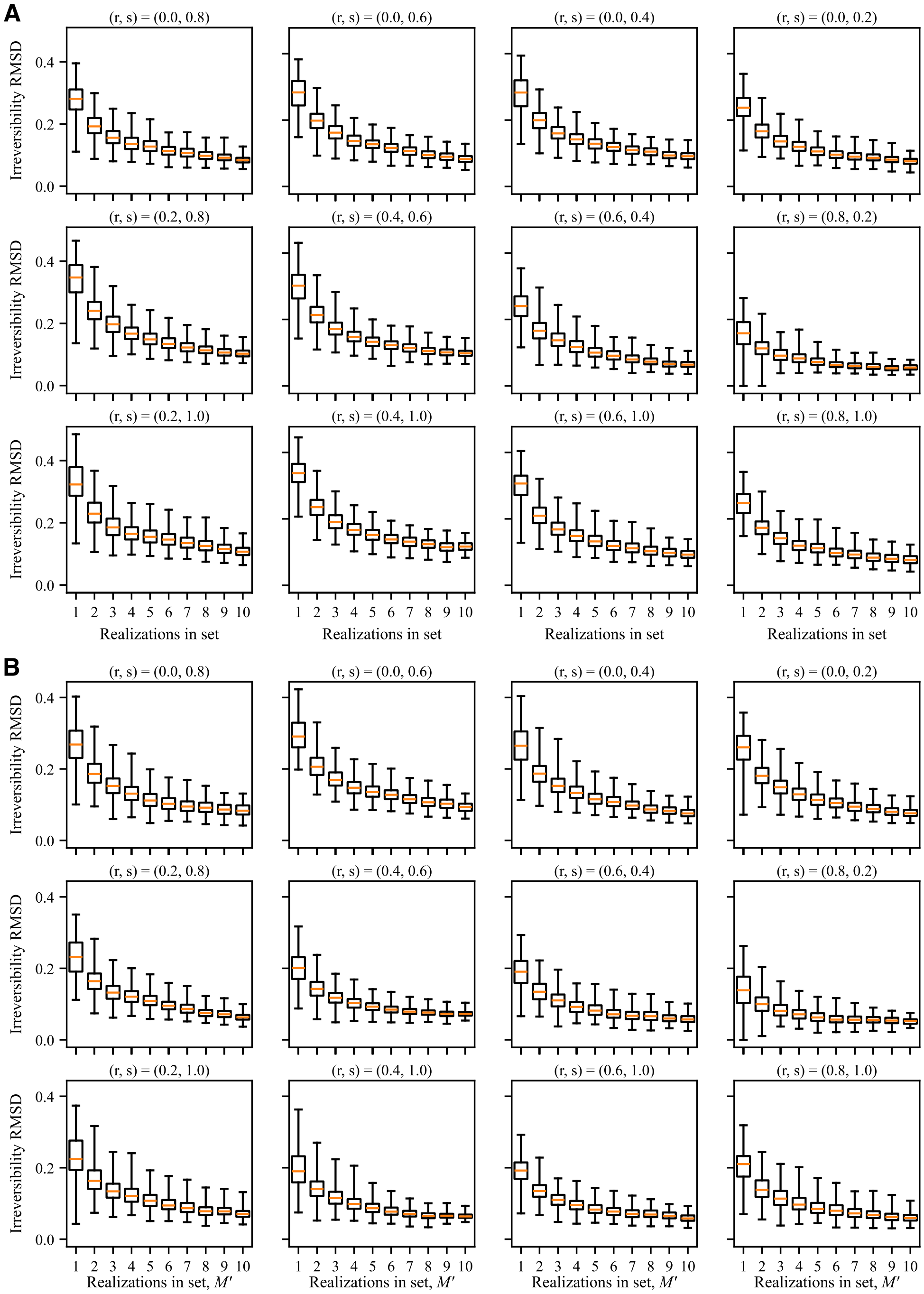

Fig. S2 Convergence of irreversibility estimated from independent sets of realizations. (**A**) Boxplots of the RMSD of the irreversibility probability across $\min(Z(M, M'), 1{,}000)$ independent pairs of realizations of the rules for the ascending input sorting (diffuse control). (**B**) Same quantities as in (A) for the descending input sorting (concentrated control). The values of the bias (s) and nestedness (r) parameters are indicated above each panel. Boxplot symbols retain their meanings from Fig. S1 (there are no fliers).

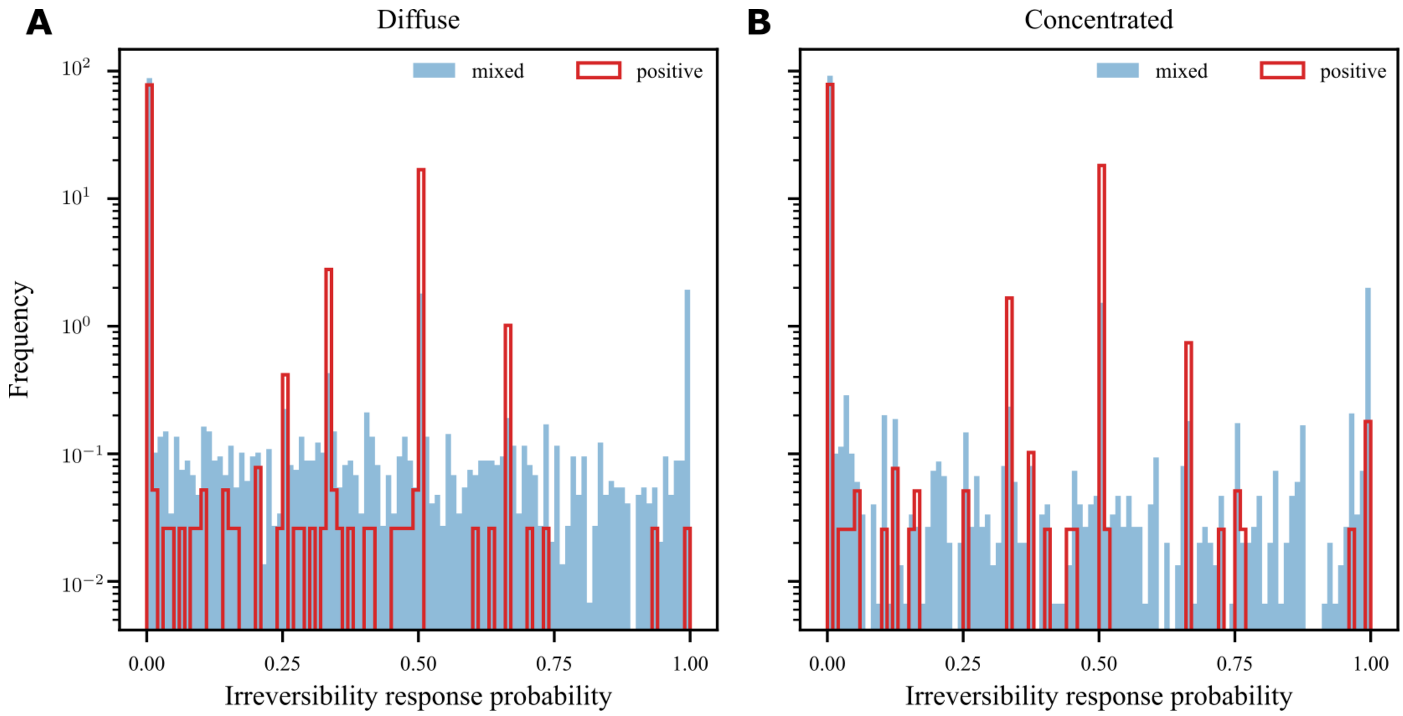

Fig. S3 Comparison of irreversible response probability depending composition of circuits in the SCC containing the irreversible response gene. (**A**) Histograms of the irreversibility response probability across all network ensemble parameters (r, s) and perturbations of *crp* under the diffuse control scenario. Genes are divided into groups by whether they belong to an SCC with only positive circuits (red) or a mixture of positive and negative circuits (blue). (**B**) Same as (A), but for the concentrated control scenario.

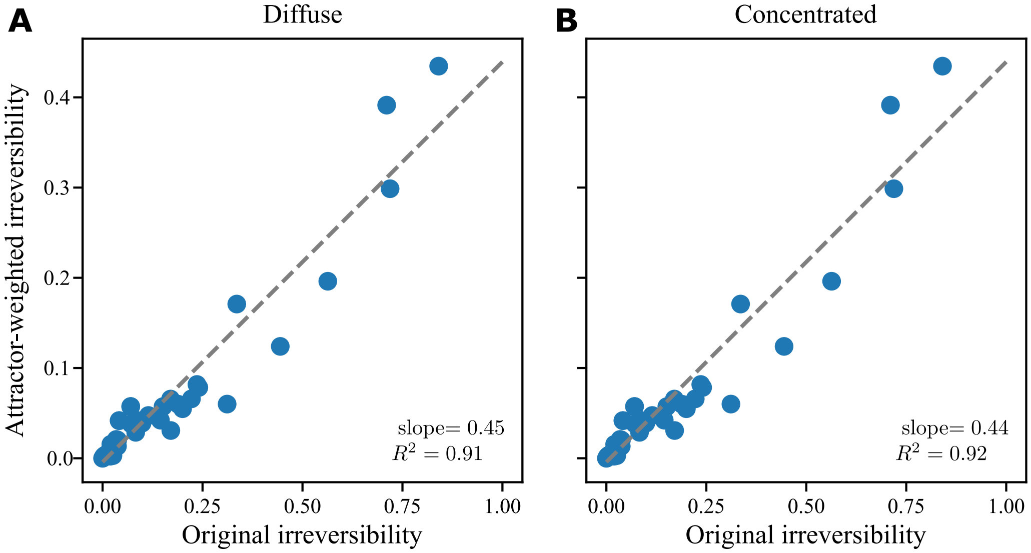

Fig. S4 Comparison of irreversibility when weighting the results by attractor basin size versus weighting them uniformly by attractor. (**A**) Scatter plot of the irreversibility of each gene averaged over perturbation types, rules realizations, and attractors in the diffuse control scenario (ascending input ordering). The gray dashed line is a linear fit to the data, whose slope indicates the quantitative change in irreversibility due to attractor weighting. The R^2 indicates the value of the coefficient of determination. (**B**) Same as (A), but for the concentrated control scenario.

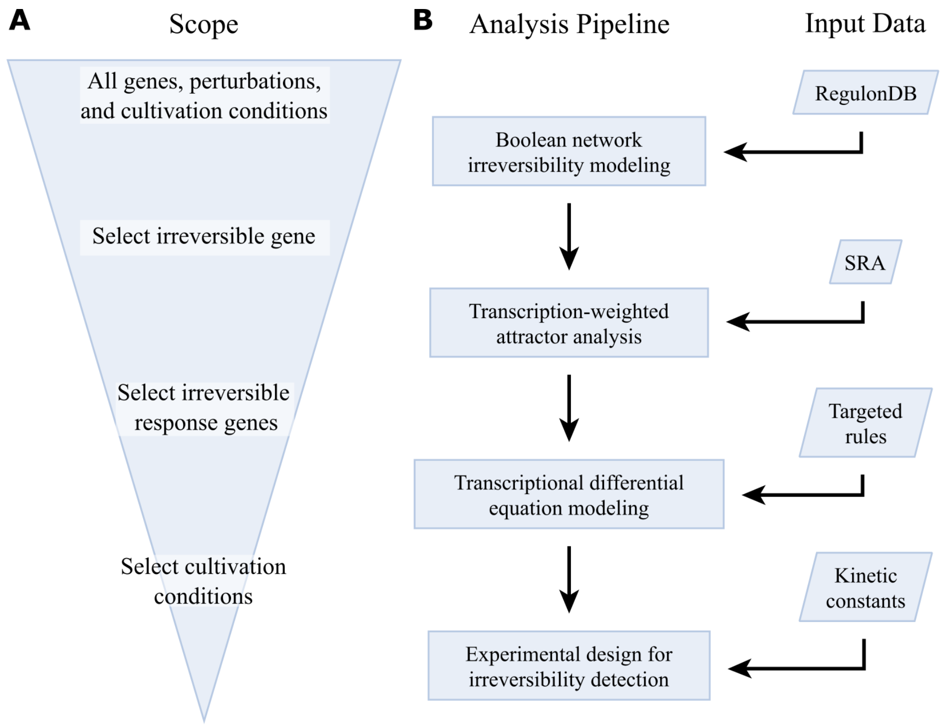

Fig. S5 Schematic for designing experiments to find irreversibility. (**A**) Diagram of how the scope is narrowed from all possible gene perturbations, irreversible response genes, and cultivation conditions to a manageable number of experimental predictions. (**B**) Analysis and input data needed to narrow the scope at each stage.

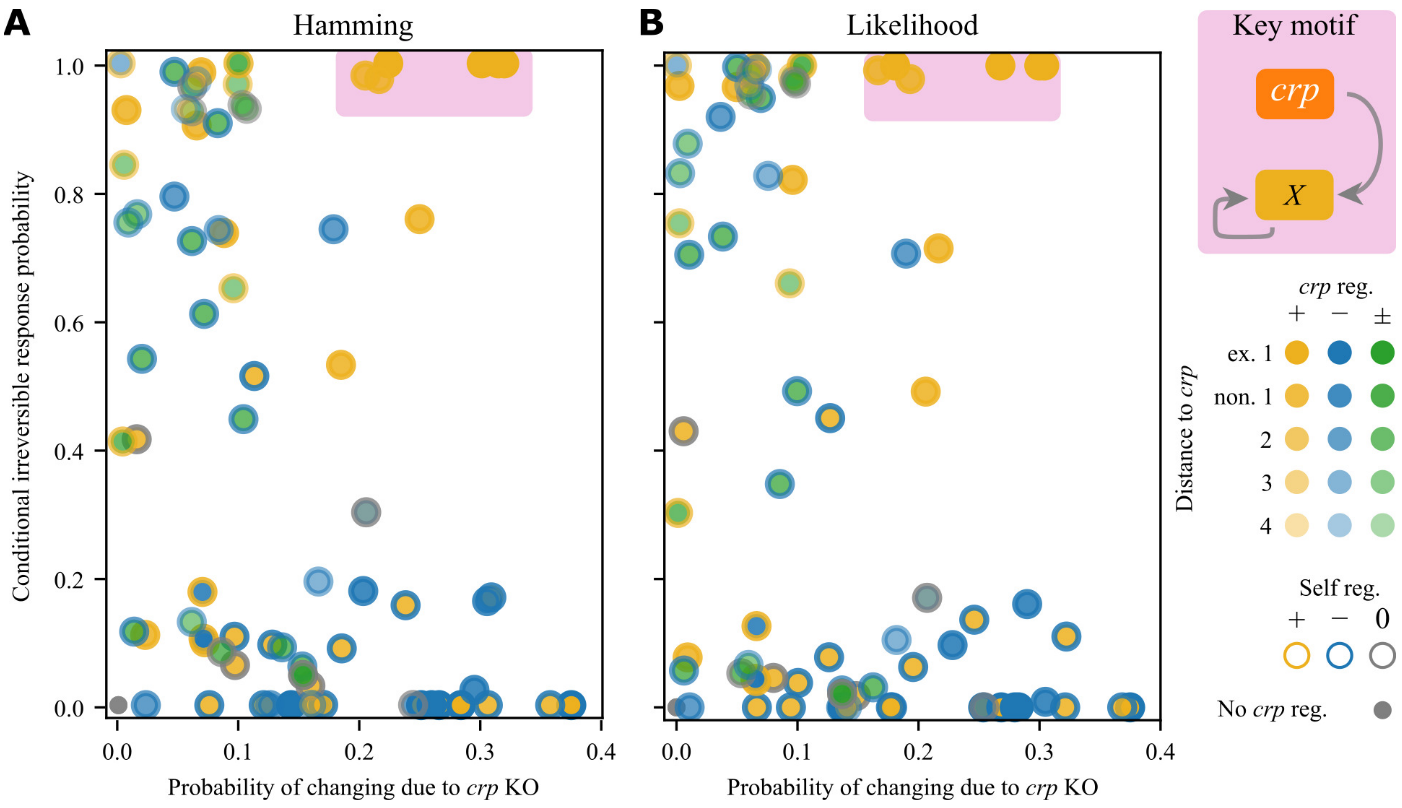

Fig. S6 Probability of irreversible response after weighting attractors by their similarity to observed states. (**A**) Probability of responding irreversibly conditioned on a gene changing in response to *crp* KO. Probabilities indicated on the vertical axis are expressed as a fraction of the corresponding values indicated on the horizontal axis. Attractors are weighted proportionally to the exponential of the negative Hamming distance (see Eq. (9)). Node face colors correspond to the sign of *crp* regulation, node edge colors correspond to the sign of self-regulation, and node opacities correspond to the distance from *crp* as summarized in the legend at right. (**B**) Same plot as in (A), when attractors are weighted by likelihood (see Eq. (10)). In both panels, the genes in the pink box belong to the key motif in which *crp* activates the gene *X* and the gene *X* activates itself.

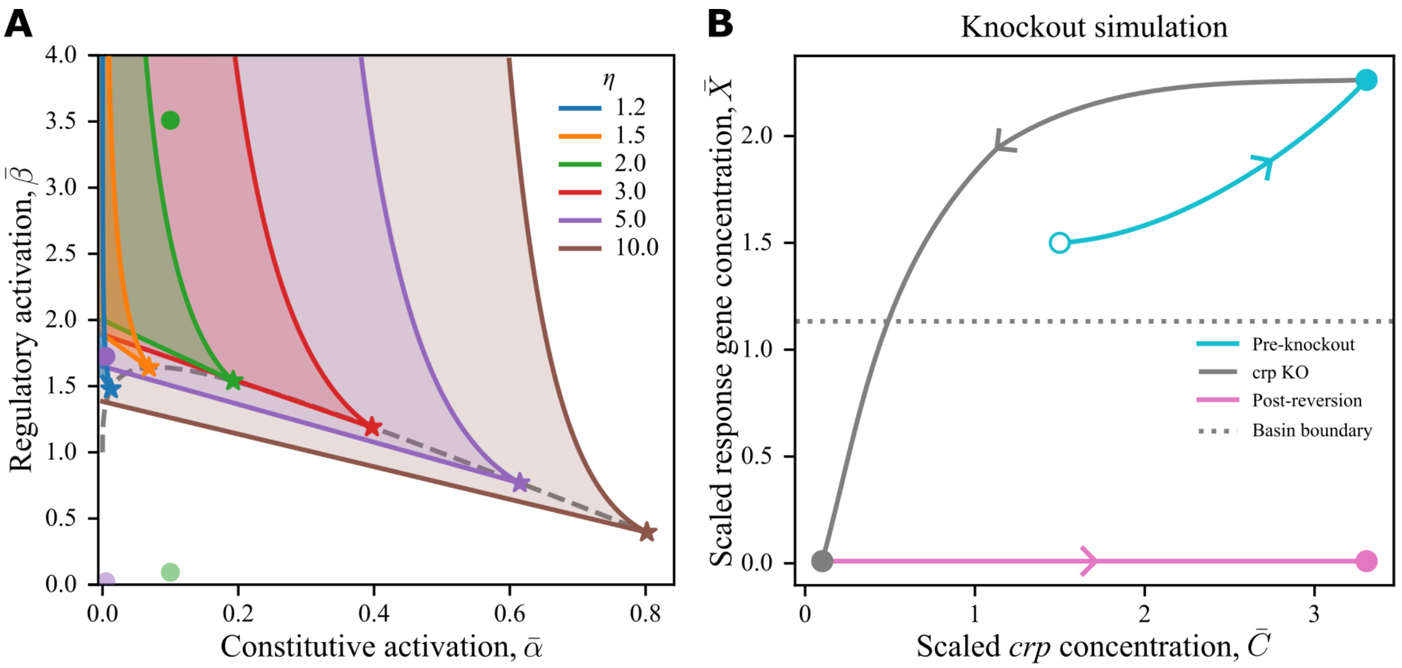

Fig. S7 Irreversibility in differential equation models of a the key motif in Fig. S6. (**A**) Multistability regions in the constituative activation–regulatory activation plane, color-coded by the values the Hill coefficient (η) indicated in the legend. Stars indicate the critical point (derived from Eqs. (21) and (22)) for each η, while the gray dashed line shows the trajectory of the critical point as η is continuously increased from 1 to 10. Circles indicate exemplar parameters for the constitutive and regulatory activation of *crp* (green, indicating $\eta_C = 2$) and the response gene (purple, indicating $\eta_X = 5$) when *crp* is active (bold) and knocked out (faded). (**B**) Concentrations of *crp* and the response gene prior to (teal), during (gray), and after *crp* KO (pink), where arrows indicate the direction of time. During each phase, the system is integrated until the system reaches the stable state. The gray dotted line indicates the basin boundary (unstable fixed point in the response-gene concentration) when *crp* is active.

Supplementary Tables

Table S1 Statistics of the origons with core size ≥ 30.

Origon root	Total size	Core size	Overlap with *phoB* core
phoB	1406	87	87
nsrR	1399	88	81
acrR	941	59	58
cpxR	550	35	33
phoP	535	32	29
slyA	476	30	29
rutR	475	31	29
basR	474	32	30
torR	468	30	29
rcdA	466	30	29
sdiA	464	30	29
sutR	463	30	29
ecpR	463	30	29
lrhA	462	30	29

Table S2 Irreversibility of genes with log-fold change > 0.5 when batch cultivated following adaptive evolution to *crp* KO.

Gene	Irreversible response probability	Shortest path from *crp*	σ_u^{mod}	k_u^+	Autoregulation	$\ln \rho_u/\langle\rho\rangle$
melR	0.22	1	$+$	2	$+$	-3.11
flhD	0.02	1	$+$	9	0	-1.72
malI	0.00	1	$+$	2	$-$	-1.65
rcsA	0.03	2	$-$	4	$+$	1.42
rhaR	0.19	1	$+$	3	$+$	-1.24
flhC	0.01	1	$+$	9	0	-1.17
tdcA	0.04	1	$+$	3	$+$	-1.14
adiY	0.03	3	$\pm$	1	0	-0.66
bglJ	0.03	3	$\pm$	2	0	-0.64
ptsG	0.02	1	$+$	6	$-$	0.62
lsrR	0.00	1	$+$	2	$-$	-0.61

Table S3 Irreversibility of genes with log-fold change > 0.5 when chemostat cultivated following adaptive evolution to *crp* KO.

Gene	Irreversible response probability	Shortest path from *crp*	σ_u^{mod}	k_u^+	Autoregulation	$\ln \rho_u / \langle \rho \rangle$
tdcA	0.04	1	+	3	+	−3.65
melR	0.22	1	+	2	+	−3.02
flhD	0.02	1	+	9	0	−2.48
aidB	0.01	3	+	2	−	2.46
rhaR	0.19	1	+	3	+	−2.38
flhC	0.01	1	+	9	0	−2.16
glcC	0.00	1	+	4	−	−2.12
gadE	0.02	1	−	10	+	1.98
lsrR	0.00	1	+	2	−	−1.71
ydeO	0.03	2	−	7	−	−1.66
fucR	0.20	1	+	2	+	−1.64
prpR	0.00	1	+	3	−	−1.64
galS	0.04	1	+	3	−	−1.59
hns	0.03	2	±	4	−	−1.51
leuO	0.02	3	±	5	+	−1.47
pdeL	0.00	NA	NA	2	+	−1.40
yeiL	0.02	3	±	3	+	−1.31
fur	<0.01	1	+	4	−	−1.23
mlc	0.01	1	−	3	−	−1.15
malI	0.00	1	+	2	−	−1.12
yjjQ	0.03	3	±	2	0	−1.09
gadW	0.02	2	±	8	−	1.08
bglJ	0.03	3	±	2	0	−1.07
evgA	0.03	3	±	2	+	−0.98
srlR	0.01	1	+	4	−	−0.96
rbsR	0.00	1	+	2	−	−0.91
cspA	0.03	2	+	2	0	−0.91
dcuR	0.04	1	+	3	0	−0.80
metR	<0.01	3	+	2	−	−0.79
fnr	0.10	2	−	3	−	−0.78
narL	0.09	3	+	1	0	0.75
gadX	0.03	1	−	13	+	0.72
lldR	0.05	4	+	2	−	−0.69
rhaS	0.19	1	+	3	+	−0.67
nhaR	0.03	3	±	2	+	0.64
fliZ	0.02	2	±	4	0	−0.59
purR	<0.01	2	−	2	−	−0.56
yqjI	0.04	3	−	2	−	0.55
cra	0.00	NA	NA	1	0	−0.55
glnG	0.00	1	+	3	−	−0.55
nac	0.03	2	±	4	−	−0.54
fhlA	0.01	3	−	2	+	0.51

REFERENCES AND NOTES

1. A. J. Koch, H. Meinhardt, Biological pattern formation: From basic mechanisms to complex structures. *Rev. Mod. Phys.* **66**, 1481–1507 (1994).

2. A. T. Winfree, *The Geometry of Biological Time* (Springer, 2001).

3. C. W. Lynn, C. M. Holmes, W. Bialek, D. J. Schwab, Emergence of local irreversibility in complex interacting systems. *Phys. Rev. E* **106**, 034102 (2022).

4. N. C. Keim, J. D. Paulsen, Z. Zeravcic, S. Sastry, S. R. Nagel, Memory formation in matter. *Rev. Mod. Phys.* **91**, 035002 (2019).

5. F. H. C. Crick, Central dogma of molecular biology. *Nature* **227**, 561–563 (1970).

6. C. Villarreal, P. Padilla-Longoria, E. R. Alvarez-Buylla, General theory of genotype to phenotype mapping: Derivation of epigenetic landscapes from *N*-node complex gene regulatory networks. *Phys. Rev. Lett.* **109**, 118102 (2012).

7. S. Huang, G. Eichler, Y. Bar-Yam, D. E. Ingber, Cell fates as high-dimensional attractor states of a complex gene regulatory network. *Phys. Rev. Lett.* **94**, 128701 (2005).

8. J. X. Zhou, S. Huang, Understanding gene circuits at cell-fate branch points for rational cell reprogramming. *Trends Genet.* **27**, 55–62 (2011).

9. J. G. T. Zañudo, R. Albert, Cell fate reprogramming by control of intracellular network dynamics. *PLoS Comput. Biol.* **11**, e1004193 (2015).

10. R. Zhu, J. M. del Rio-Salgado, J. Garcia-Ojalvo, M. B. Elowitz, Synthetic multistability in mammalian cells. *Science* **375**, eabg9765 (2022).

11. C. López-Otín, M. A. Blasco, L. Partridge, M. Serrano, G. Kroemer, The hallmarks of aging. *Cell* **153**, 1194–1217 (2013).

12. S. He, N. E. Sharpless, Senescence in health and disease. *Cell* **169**, 1000–1011 (2017).

13. L. Shapiro, N. Agabian-Keshishian, I. Bendis, Bacterial differentiation. *Science* **173**, 884–892 (1971).

14. J.-W. Veening, W. K. Smits, O. P. Kuipers, Bistability, epigenetics, and bet-hedging in bacteria. *Annu. Rev. Microbiol.* **62**, 193–210 (2008).

15. E. M. Ozbudak, M. Thattai, H. N. Lim, B. I. Shraiman, A. van Oudenaarden, Multistability in the lactose utilization network of *Escherichia coli*. *Nature* **427**, 737–740 (2004).

16. M. K. Prajapat, K. Jain, S. Saini, Control of MarRAB operon in *Escherichia coli* via autoactivation and autorepression. *Biophys. J.* **109**, 1497–1508 (2015).

17. A. Santos-Zavaleta, M. Sánchez-Pérez, H. Salgado, D. A. Velázquez-Ramírez, S. Gama-Castro, V. H. Tierrafría, S. J. W. Busby, P. Aquino, X. Fang, B. O. Palsson, J. E. Galagan, J. Collado-Vides, A unified resource for transcriptional regulation in *Escherichia coli* K-12 incorporating high-throughput-generated binding data into RegulonDB version 10.0. *BMC Biol.* **16**, 91 (2018).

18. G. Balazsi, A.-L. Barabasi, Z. N. Oltvai, Topological units of environmental signal processing in the transcriptional regulatory network of *Escherichia coli*. *Proc. Natl. Acad. Sci. U.S.A.* **102**, 7841–7846 (2005).

19. A. Samal, S. Jain, The regulatory network of *E. coli* metabolism as a Boolean dynamical system exhibits both homeostasis and flexibility of response. *BMC Syst. Biol.* **2**, 21 (2008).

20. N. Azimi-Tafreshi, S. N. Dorogovtsev, J. F. F. Mendes, Core organization of directed complex networks. *Phys. Rev. E* **87**, 032815 (2013).

21. S. A. Kauffman, *The Origins of Order: Self-organization and Selection in Evolution* (Oxford Univ. Press, 1993).

22. R.-S. Wang, A. Saadatpour, R. Albert, Boolean modeling in systems biology: An overview of methodology and applications. *Phys. Biol.* **9**, 055001 (2012).

23. Q. He, M. Macauley, Stratification and enumeration of Boolean functions by canalizing depth. *Phys. D* **314**, 1–8 (2016).

24. S. E. Harris, B. K. Sawhill, A. Wuensche, S. Kauffman, A model of transcriptional regulatory networks based on biases in the observed regulation rules. *Complexity* **7**, 23–40 (2002).

25. S. Kauffman, C. Peterson, B. Samuelsson, C. Troein, Random Boolean network models and the yeast transcriptional network. *Proc. Natl. Acad. Sci. U.S.A.* **100**, 14796–14799 (2003).

26. A. A. Moreira, L. A. N. Amaral, Canalizing Kauffman networks: Nonergodicity and its effect on their critical behavior. *Phys. Rev. Lett.* **94**, 218702 (2005).

27. W. V. Quine, A way to simplify truth functions. *Am. Math. Mon.* **62**, 627–631 (1955).

28. E. J. McCluskey, Minimization of Boolean functions. *Bell Syst. Tech. J.* **35**, 1417–1444 (1956).

29. A. Pomerance, E. Ott, M. Girvan, W. Losert, The effect of network topology on the stability of discrete state models of genetic control. *Proc. Natl. Acad. Sci. U.S.A.* **106**, 8209–8214 (2009).

30. S. Squires, A. Pomerance, M. Girvan, E. Ott, Stability of Boolean networks: The joint effects of topology and update rules. *Phys. Rev. E* **90**, 022814 (2014).

31. S. Tripathi, D. A. Kessler, H. Levine, Biological networks regulating cell fate choice are minimally frustrated. *Phys. Rev. Lett.* **125**, 088101 (2020).

32. S. Tripathi, D. A. Kessler, H. Levine, Minimal frustration underlies the usefulness of incomplete regulatory network models in biology. *Proc. Natl. Acad. Sci. U.S.A.* **120**, e2216109120 (2023).

33. B. Alexander, A. Pushkar, M. Girvan, Phase transitions and assortativity in models of gene regulatory networks evolved under different selection processes. *J. R. Soc. Interface* **18**, 20200790 (2021).

34. S. S. Shen-Orr, R. Milo, S. Mangan, U. Alon, Network motifs in the transcriptional regulation network of *Escherichia coli*. *Nat. Genet.* **31**, 64–68 (2002).

35. T. I. Lee, N. J. Rinaldi, F. Robert, D. T. Odom, Z. Bar-Joseph, G. K. Gerber, N. M. Hannett, C. T. Harbison, C. M. Thompson, I. Simon, J. Zeitlinger, E. G. Jennings, H. L. Murray, D. B. Gordon, B. Ren, J. J. Wyrick, J.-B. Tagne, T. L. Volkert, E. Fraenkel, D. K. Gifford, R. A. Young, Transcriptional regulatory networks in *Saccharomyces cerevisiae*. *Science* **298**, 799–804 (2002).

36. L. A. Boyer, T. I. Lee, M. F. Cole, S. E. Johnstone, S. S. Levine, J. P. Zucker, M. G. Guenther, R. M. Kumar, H. L. Murray, R. G. Jenner, D. K. Gifford, D. A. Melton, R. Jaenisch, R. A. Young, Core transcriptional regulatory circuitry in human embryonic stem cells. *Cell* **122**, 947–956 (2005).

37. E. Dubrova, M. Teslenko, A SAT-based algorithm for finding attractors in synchronous boolean networks. *IEEE/ACM Trans. Comput. Biol. Bioinform.* **8**, 1393–1399 (2011).

38. É. Remy, P. Ruet, D. Thieffry, Graphic requirements for multistability and attractive cycles in a Boolean dynamical framework. *Adv. Appl. Math.* **41**, 335–350 (2008).

39. D. Angeli, J. E. Ferrell, E. D. Sontag, Detection of multistability, bifurcations, and hysteresis in a large class of biological positive-feedback systems. *Proc. Natl. Acad. Sci. U.S.A.* **101**, 1822–1827 (2004).

40. G. Craciun, C. Pantea, E. D. Sontag, *Design and Analysis of Biomolecular Circuits: Engineering Approaches to Systems and Synthetic Biology*, H. Koeppl, G. Setti, M. di Bernardo, D. Densmore, Eds. (Springer New York, 2011), pp. 63–72.

41. A. Pal, M. S. Iyer, S. Srinivasan, A. S. Narain Seshasayee, K. V. Venkatesh, Global pleiotropic effects in adaptively evolved *Escherichia coli* lacking CRP reveal molecular mechanisms that define the growth physiology. *Open Biol.* **12**, 210206 (2022).

42. D. M. Wittmann, J. Krumsiek, J. Saez-Rodriguez, D. A. Lauffenburger, S. Klamt, F. J. Theis, Transforming Boolean models to continuous models: Methodology and application to T-cell receptor signaling. *BMC Syst. Biol.* **3**, 98 (2009).

43. L. S. Qi, M. H. Larson, L. A. Gilbert, J. A. Doudna, J. S. Weissman, A. P. Arkin, W. A. Lim, Repurposing CRISPR as an RNA-guided platform for sequence-specific control of gene expression. *Cell* **152**, 1173–1183 (2013).

44. T. Afroz, K. Biliouris, Y. Kaznessis, C. L. Beisel, Bacterial sugar utilization gives rise to distinct single-cell behaviours. *Mol. Microbiol.* **93**, 1093–1103 (2014).

45. H. H. Chang, M. Hemberg, M. Barahona, D. E. Ingber, S. Huang, Transcriptome-wide noise controls lineage choice in mammalian progenitor cells. *Nature* **453**, 544–547 (2008).

46. D. Schultz, P. G. Wolynes, E. B. Jacob, J. N. Onuchic, Deciding fate in adverse times: Sporulation and competence in *Bacillus subtilis*. *Proc. Natl. Acad. Sci. U.S.A.* **106**, 21027–21034 (2009).

47. M. Sasai, Y. Kawabata, K. Makishi, K. Itoh, T. P. Terada, Time scales in epigenetic dynamics and phenotypic heterogeneity of embryonic stem cells. *PLoS Comput. Biol.* **9**, e1003380 (2013).

48. S. Tripathi, H. Levine, M. K. Jolly, The physics of cellular decision making during epithelial–mesenchymal transition. *Annu. Rev. Biophys.* **49**, 1–18 (2020).

49. D. K. Wells, W. L. Kath, A. E. Motter, Control of stochastic and induced switching in biophysical networks. *Phys. Rev. X* **5**, 031036 (2015).

50. J. G. T. Zañudo, G. Yang, R. Albert, Structure-based control of complex networks with nonlinear dynamics. *Proc. Natl. Acad. Sci. U.S.A.* **114**, 7234–7239 (2017).

51. E. Sullivan, M. Harris, A. Bhatnagar, E. Guberman, I. Zonfa, E. Ravasz Regan, Boolean modeling of mechanosensitive epithelial to mesenchymal transition and its reversal. *iScience* **26**, 106321 (2023).

52. J. E. M. Hornos, D. Schultz, G. C. P. Innocentini, J. Wang, A. M. Walczak, J. N. Onuchic, P. G. Wolynes, Self-regulating gene: An exact solution. *Phys. Rev. E* **72**, 051907 (2005).

53. A. M. Walczak, J. N. Onuchic, P. G. Wolynes, Absolute rate theories of epigenetic stability. *Proc. Natl. Acad. Sci. U.S.A.* **102**, 18926–18931 (2005).

54. G. Tkačik, A. M. Walczak, W. Bialek, Optimizing information flow in small genetic networks. III. A self-interacting gene. *Phys. Rev. E* **85**, 041903 (2012).

55. M. Assaf, E. Roberts, Z. Luthey-Schulten, N. Goldenfeld, Extrinsic noise driven phenotype switching in a self-regulating gene. *Phys. Rev. Lett.* **111**, 058102 (2013).

56. K. Zhang, M. Sasai, J. Wang, Eddy current and coupled landscapes for nonadiabatic and nonequilibrium complex system dynamics. *Proc. Natl. Acad. Sci. U.S.A.* **110**, 14930–14935 (2013).

57. B. Bhattacharyya, J. Wang, M. Sasai, Stochastic epigenetic dynamics of gene switching. *Phys. Rev. E* **102**, 042408 (2020).

58. C. Müssel, M. Hopfensitz, H. A. Kestler, BoolNet—An R package for generation, reconstruction and analysis of Boolean networks. *Bioinformatics* **26**, 1378–1380 (2010).

59. T. Barrett, S. E. Wilhite, P. Ledoux, C. Evangelista, I. F. Kim, M. Tomashevsky, K. A. Marshall, K. H. Phillippy, P. M. Sherman, M. Holko, A. Yefanov, H. Lee, N. Zhang, C. L. Robertson, N. Serova, S. Davis, A. Soboleva, NCBI GEO: Archive for functional genomics data sets—Update. *Nucleic Acids Res.* **41**, D991–D995 (2012).

60. B. Efron, R. Tibshirani, The bootstrap method for assessing statistical accuracy. *Behav. Ther.* **12**, 1–35 (1985).

61. F. Mori, A. Mochizuki, Expected number of fixed points in boolean networks with arbitrary topology. *Phys. Rev. Lett.* **119**, 028301 (2017).

62. J. C. Rozum, J. G. T. Zañudo, X. Gan, D. Deritei, R. Albert, Parity and time reversal elucidate both decision-making in empirical models and attractor scaling in critical Boolean networks. *Sci. Adv.* **7**, abf8124 (2021).

63. X. Gan, R. Albert, General method to find the attractors of discrete dynamic models of biological systems. *Phys. Rev. E* **97**, 042308 (2018).

64. S. S. Justice, A. Harrison, B. Becknell, K. M. Mason, Bacterial differentiation, development, and disease: Mechanisms for survival. *FEMS Microbiol. Lett.* **360**, 1–8 (2014)

65. W. K. Smits, O. P. Kuipers, J.-W. Veening, Phenotypic variation in bacteria: The role of feedback regulation. *Nat. Rev. Microbiol.* **4**, 259–271 (2006)

66. R. McClure, D. Balasubramanian, Y. Sun, M. Bobrovskyy, P. Sumby, C. A. Genco, C. K. Vanderpool, B. Tjaden, Computational analysis of bacterial RNA-Seq data. *Nucleic Acids Res.* **41**, e140 (2013).
